# Open source arc analyzer: Multi-sensor monitoring of wire arc additive manufacturing

**DOI:** 10.1016/j.ohx.2020.e00137

**Published:** 2020-09-02

**Authors:** Adam M. Pringle, Shane Oberloier, Aliaksei L. Petsiuk, Paul G. Sanders, Joshua M. Pearce

**Affiliations:** a Department of Materials Science & Engineering, Michigan Technological University, Houghton, MI 49931-1295, USA; b Department of Electrical & Computer Engineering, Michigan Technological University, Houghton, MI 49931-1295, USA; c Department of Electronics and Nanoengineering, School of Electrical Engineering, Aalto University, Espoo, Finland

**Keywords:** 3-D printing, gas metal arc weld, GMAW, metal inert gas welding, MIG welding, additive manufacturing, metal printing, Open-source hardware, RepRap, Welder, Welding, Metal 3-D printing, low cost metal 3-D printer, open-source metal 3-D printer, GMAW 3-D printing, Wire Arc Additive Manufacturing, WAAM

## Abstract

Low-cost high-resolution metal 3-D printing remains elusive for the scientific community. Low-cost gas metal arc wire (GMAW)-based 3-D printing enables wire arc additive manufacturing (WAAM) for near net shape applications, but has limited resolution due to the complexities of the arcing process. To begin to monitor and thus control these complexities, the initial designs of the open source GMAW 3-D printer have evolved to include current and voltage monitoring. Building on this prior work, in this study, the design, fabrication and use of the open source arc analyzer is described. The arc analyzer is a multi-sensor monitoring system for quantifying the processing during WAAM, which includes voltage, current, sound, light intensity, radio frequency, and temperature data outputs. The open source arc analyzer is tested here on aluminum WAAM by varying wire feed rate and measuring the resultant changes in the sensor data. Visual inspection and microstructural analysis of the printed samples looking for the presence of porosity are used as the physical indicators of quality. The value of the sensors was assessed and the most impactful sensors were found to be the light and radio frequency sensors, which showed arc extinction events and a characteristic “good weld” peak frequency.

## Graphical abstract

**Figure:**
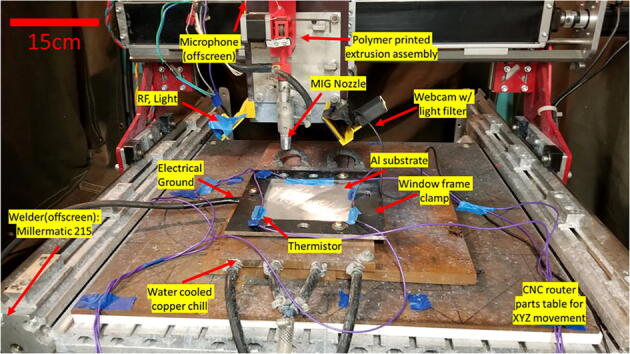

**Specifications table:** 

Hardware name	*Arc Analyzer*
Subject area	• Engineering and Material Science
Hardware type	• Measuring physical properties and in-lab sensors
Open Source License	*GNU General Public License v. 3*
Cost of Hardware	*$128*
Source File Repository	https://osf.io/y85j2/

## 1 Hardware in context

The high costs and complexity of commercial metal additive manufacturing (AM) restricts access to the technology for distributed manufacturing and rapid prototyping in small and medium enterprises (SMEs), fablabs, research centers, many labs and individual makers [1]. The self-Replicating Rapid-prototyper (RepRap) community [2], [3], [4], has developed an open-source metal 3-D printer with a gas metal arc welding (GMAW)-based print head, which reduces the costs of metal 3-D printers to less than $1200 [5] and thus greatly expands accessibility of metal AM. GMAW is an arc welding process where a consumable electrode metal wire is melted via an electric arc and combined with a base material. The metal wire is fed continuously through the generated arc, surrounded by protective cover gas and into a dynamic weld pool while the nozzle is translated over a base plate or part. If used with successive layer depositions GMAW becomes wire arc additive manufacturing (WAAM).

Researchers using these open source machines have found other ways to reduce costs including reusing substrates that reduce the concomitant embodied energy, post processing time and the environment impact of manufacturing [6], [7]. Previous work has helped the refinement of the technique including 3-D weld deposit-based process [8], [9], [10], [11], thermal properties [12], [13], [14], [15], 3-D print head path planning and slicing [16], [17], [18], [19], [20], bead-width control [21], [22], structural-property relationship and mechanical properties [23], [24], [25], and ways to support geometries to allow for overhangs [26]. This research has shown that GMAW-based metal 3-D printing produces solids with low porosity and good adhesion between layers, but the minimum feature size is greater (~0.5 mm for steel and ~2 mm for aluminum) than those of conventional material extrusion-based polymer 3-D printing as well as powder-based metal 3-D printing techniques. These large track widths enable WAAM to have high metal deposition rates and has the potential to be a suitable candidate for replacing or augmenting current manufacturing methods especially when a near net shape is allowed [27].

To push this technology to applications beyond those only requiring a low resolution [1] process, monitoring and control of the arc are necessary. This is because of the volatile nature of metal liquefying and being deposited during welding creates inconsistencies and weld quality problems [28]. For wire arc methods, weld quality problems arise due to poor arc stability or noise factors such as contamination or environmental conditions [29]. Arc stability is dependent on uniform material transfer, efficient gas coverage, minimal arc length variation and effective electrical grounding [29]. Contamination can take the form of hydrocarbons on wire or base material that is improperly stored or cleaned [28]. Hydrocarbons have low melting or decomposition temperatures and if exposed to the high temperature of the plasma arc will lead to arc stability degradation [28]. Adverse environmental conditions could include non-inert gas entering the welding environment and decreasing cover gas efficiency or having greater than 2.25 m/s crosswind. Any of these problems would propagate through successive layers in a WAAM printed part.

To begin to monitor and thus control these complexities, the initial designs of the open source GMAW 3-D printer have evolved to include current and voltage monitoring [30], [31]. Building on this prior work, in this study, the design, fabrication and use of the open source arc analyzer is described. The arc analyzer is a multi-sensor monitoring system for quantifying the processing during WAAM, which includes: voltage, current, sound, light intensity, radio frequency, and temperature data outputs. The open source arc analyzer is tested here on aluminum WAAM by varying metal wire feed rate and measuring the resultant changes in the sensor data. Statistical validation is done on the observed data and microstructural analysis on the printed samples. The presence of porosity is used as the physical indicator of quality. This approach provides guidance for further improving the WAAM process as it finds differences in sensor data associated with physical and dimensional indicators of quality.

## 2 Hardware description

The open source arc analyzer is a multi-sensor monitoring system for quantifying the processing during WAAM, which includes: voltage, current, sound, light intensity, radio frequency, and temperature data outputs. The design incorporates previous work by Pinar et al, 2015 [30] for monitoring voltage and current directly from the welder power source. The arc analyzer is intended to allow greater insight into the physical aspects of the WAAM process and provide quantitative data to improve 3-D printed part quality. The custom printed circuit board (PCB) uses low-cost common electrical components for fabrication allowing for local or online sourcing. The arc analyzer is paired with two polyethylene terephthalate glycol-modified (PETG) polymer 3-D printed parts: a protective 3-D printed casing and a sensor holder to position the radio frequency and light sensors. The arc analyzer enables researchers to:

• Rapidly focus on determining optimal print parameters for GMAW-based 3-D printing.
• Monitor changes in optimal print parameters based on geometry, material or processing conditions.
• Provide monitoring to enable eventual feedback and in situ correction of 3-D prints.

## 3 Design files

**Table:** Design files summary

Design file name	File type	Open source license	Location of the file
Nanodaq case Bottom.stl	STL	GNU GPL v3	https://osf.io/y85j2/
Nanodaq case Top.stl	STL	GNU GPL v3	https://osf.io/y85j2/
Nanodaq case assembly.scad	SCAD	GNU GPL v3	https://osf.io/y85j2/
Sensor holder base.stl	STL	GNU GPL v3	https://osf.io/y85j2/
Sensor clamp.stl	STL	GNU GPL v3	https://osf.io/y85j2/
Sensor holder assembly.scad	SCAD	GNU GPL v3	https://osf.io/y85j2/
Antenna.stl	STL	GNU GPL v3	https://osf.io/y85j2/
Antenna.scad	SCAD	GNU GPL v3	https://osf.io/y85j2/
Arc_Analyzer.pdf	PDF	GNU GPL v3	https://osf.io/y85j2/
Arc_Analyzer.ino	INO	GNU GPL v3	https://osf.io/y85j2/
Arc_Analyzer_Data_Acquisition_and_Processing.ipynb	IPYB	GNU GPL v3	https://osf.io/y85j2/
Single line.gcode	GCODE	GNU GPL v3	https://osf.io/y85j2/

### 3.1 File descriptions

The nanodaq case bottom and top files refer to the casing meant to protect the arc analyzer during operation. They were printed in PETG on a free and open source Lulzbot TAZ 6 (Aleph Objects, Loveland, CO). The sensor holder base and clamp files are custom designed to hold sensors in constant relative position to the plasma arc. The antenna file is the scaffolding for the wrapped wire that makes up the radio frequency sensor. The arc analyzer pdf and ino files refer to the electronic schematic and Arduino coding, respectively. Lastly, the data acquisition and processing ipynb file uses python via the Jupyter Notebook open-sourced web application.

## 4 Bill of materials

.

## 5 Build instructions

### 5.1 Instructions for assembly breakdown

1. 3-D print all of the .STL file parts in Table 1, on a fused filament fabrication (FFF) 3-D printer with a sufficiently sized print bed (270 × 270 × 40 mm minimum for nanodaq casing). The printed parts do not undergo any movement during use, nor significant mechanical stress. The recommended settings in Lulzbot Cura (version 3.6.8) for a given polymer, in this case PETG through Lulzbot, should provide adequate quality. The casing was designed to be press fit top and bottom. The tools necessary for construction are listed in Table 3

**Table 1:** Rendering of 3-D printable files.

STL File	Rendering in Cura
Nanodaq case Bottom.stl	
Nanodaq case Top.stl	
Sensor holder base.stl	
Sensor clamp.stl	
Antenna.stl

**Table 2:** Bill of materials.

Component	Number per PCB	Description	Number to order	Cost per unit ($)	Total cost ($)	Source of materials
Arduino Nano v3.x	1		1	$22.00	$22.00	https://www.digikey.com/products/en?mpart=A000005&v=1050
SMD 0.1uF ceramic capacitor 50 V	25	box of 10	3	$1.75	$5.25	https://www.digikey.com/product-detail/en/yageo/CC1206JRX7R9BB104/311–1435-1-ND/2833741
THT 10uF electrolytic capacitor 50 V	6	box of 10	1	$1.91	$1.91	https://www.digikey.com/product-detail/en/nichicon/UVZ1H100MDD1TA/493–16211-1-ND/6556102
Zener Diode, 5.1 V, 1 W	12	box of 10	1	$2.02	$2.02	https://www.digikey.com/product-detail/en/on-semiconductor/1N4733ATR/1N4733AFSCT-ND/1532752
LED, green	1		1	$0.23	$0.23	https://www.digikey.com/product-detail/en/cree-inc/C566C-GFF-CX0Y0892/C566C-GFF-CX0Y0892CT-ND/6138564
LED, red	1		1	$0.14	$0.14	https://www.digikey.com/short/q75wh9
Terminal Block, 2 Pos	13	box of 10	2	$4.14	$8.28	https://www.digikey.com/short/q7wbhd
Header Male 2	12	32 positions	1	$0.79	$0.79	https://www.digikey.com/products/en?keywords=PRPC032SAAN-RC
Header Male 3	30	32 positions	4	$0.79	$3.16	https://www.digikey.com/products/en?keywords=PRPC032SAAN-RC
Bipolar Transistor NPN, 50 V 5A TIP120	6	box of 10	1	$5.66	$5.66	https://www.digikey.com/short/q75wn4
SMD 10 K ohm resistor 1/4W	14	box of 10	2	$0.71	$1.42	https://www.digikey.com/product-detail/en/yageo/RC1206FR-0710KL/311–10.0KFRCT-ND/731430
SMD 1 K ohm resistor 1/4W	24	box of 10	3	$0.71	$2.13	https://www.digikey.com/product-detail/en/yageo/RC1206FR-071KL/311–1.00KFRCT-ND/731334
SMD 4 K7 ohm resistor 1/4W	2	box of 10	1	$0.71	$0.71	https://www.digikey.com/product-detail/en/yageo/RC1206FR-074K7L/311–4.70KFRCT-ND/731834
THT 10 K ohm trimmer potentiometer 25 turn	18	box of 10	2	$10.61	$21.22	https://www.digikey.com/short/qt5j3q
SENSOR CURRENT HALL 50A AC/DC	6	box of 5 + 1	6	$6.30	$37.80	https://www.digikey.com/product-detail/en/allegro-microsystems-llc/ACS770LCB-050B-PFF-T/620–1541-5-ND/4473980
IC RTC CLK/CALENDAR I2C 8-DIP	1		1	$4.07	$4.07	https://www.digikey.com/product-detail/en/maxim-integrated/DS1307-/DS1307--ND/956883
Dual Op Amp 6 V 1MHZ (Rail to Rail) MCP6002-I/P	6		6	$0.33	$1.98	https://www.digikey.com/short/q7whcw
IC TRNSLTR BIDIRECTIONAL 14TSSOP	1		1	$1.32	$1.32	https://www.digikey.com/product-detail/en/texas-instruments/TXB0104PWR/296–21929-1-ND/1629282
32.768 kHz Crystal	1		1	$0.21	$0.21	https://www.digikey.com/short/qt58hr
CMA-4544PF-W Mic	1	box of 10	1	$6.58	$6.58	https://www.digikey.com/product-detail/en/cui-devices/CMA-4544PF-W/102–1721-ND/1869981
M3 X 10 Screw	4	box of 100	1	$7.62	$7.62	https://www.mcmaster.com/91290a115

**Table 3:** Recommended tools for building the Arc Analyzer.

**Tool**	**Usage**
Wire strippers	Wiring of sensors to Arc Analyzer
Soldering iron	Arc Analyzer assembly
Heat gun	Shrink tubing around wiring
Screwdriver	Securing Arc Analyzer to casing
2. Using the gerber files from (https://osf.io/y85j2/) [32], upload them to https://jlcpcb.com/ and order the circuit board (see Table 2).
3. Build and solder the Arc Analyzer circuit using Fig. 1 as a schematic with reference to the components in the bill of materials. Fig. 2 shows the completed circuits for the microphone, radio frequency (antenna), light (photoresistor), temperature (thermistor), current, and voltage sections. Fig. 3 shows the wiring for the thermistor connections to the six digital I/O.

**Fig. 1:**
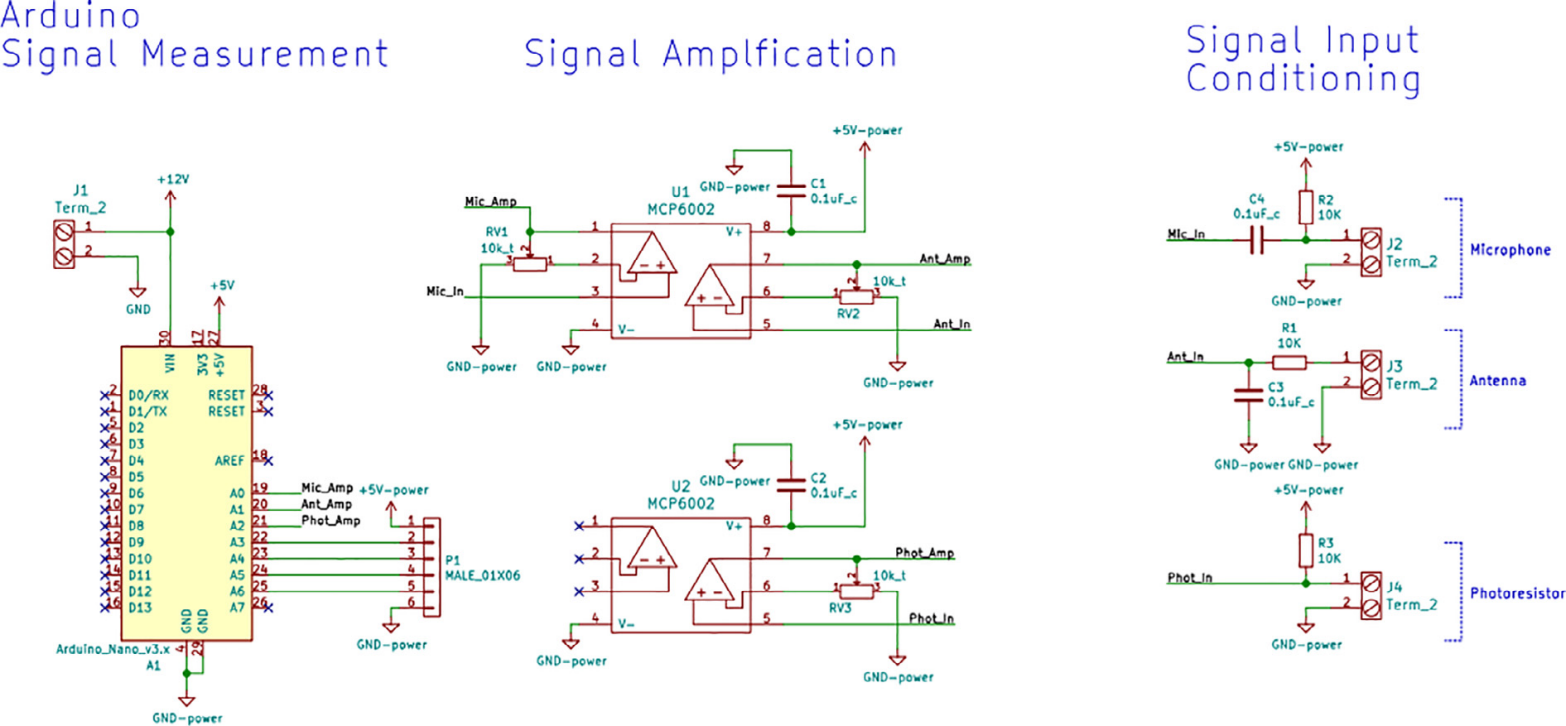
Arc Analyzer electrical schematics.

**Fig. 2:**
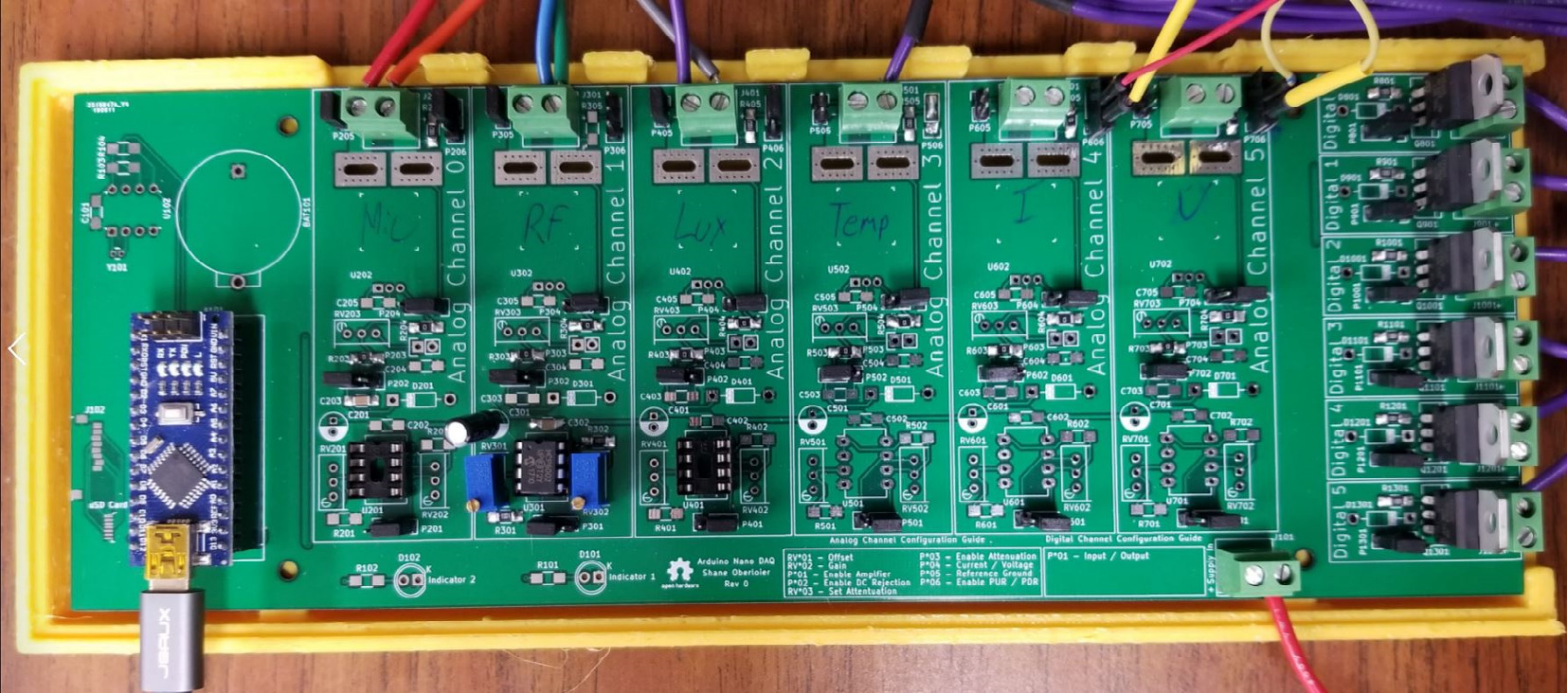
Arc Analyzer board with completed sensor circuits.

**Fig. 3:**
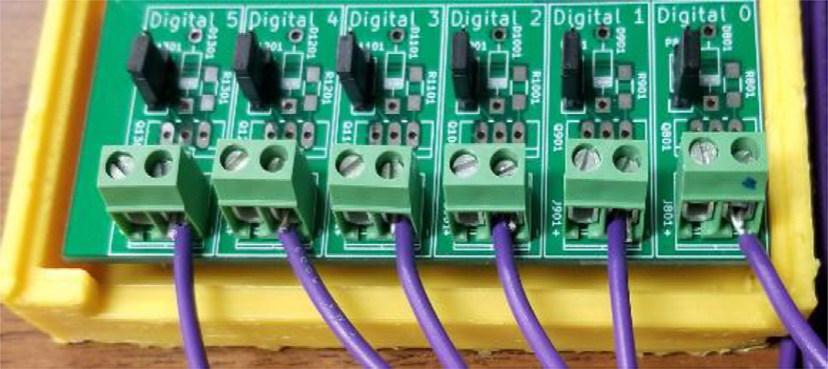
Digital I/O close up with six thermistors connected.
4. Solder and assemble all sensors as seen in Fig. 4 and wire into their corresponding channels on the main board.

**Fig. 4:**
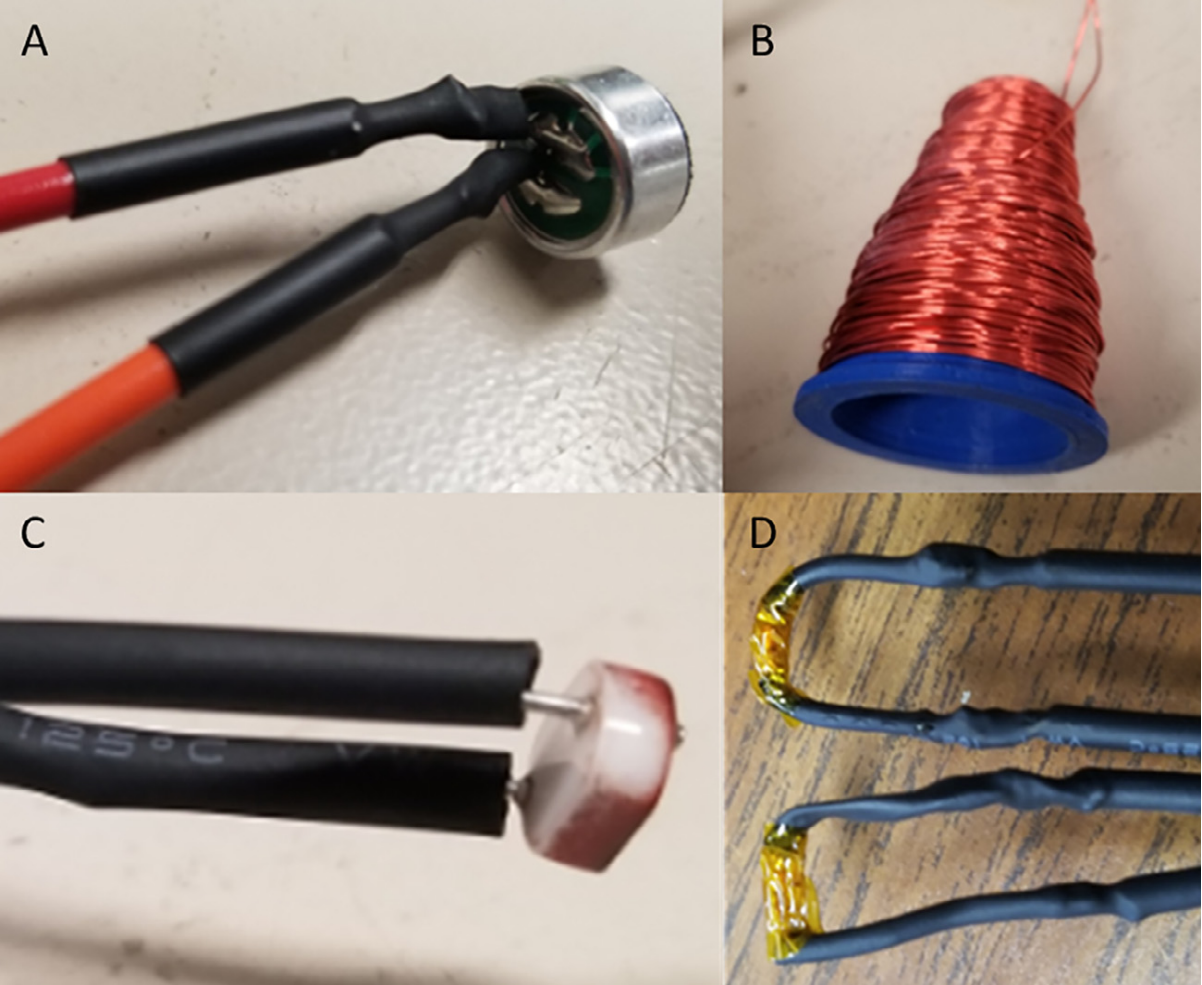
(A) microphone, (B) radio frequency antenna, (C) photoresistor, and (D) thermistors sensors used with the Arc Analyzer.
5. Build the voltage and current sensor board [30] seen in Fig. 5 and wire into the Arc Analyzer.

**Fig. 5:**
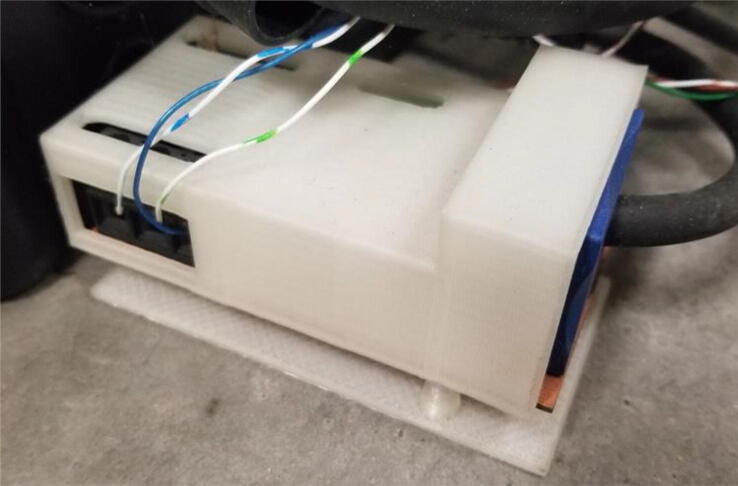
PLA casing covering voltage and current circuit attached around welding electrode constructed from Pinar et al. [30].
6. Download the Arduino IDE software (https://www.arduino.cc/) and plug in the Arduino nano. Open the Arc_Analyzer.ino file from the above design files summary. Make sure that the correct port is selected and the atmega328P processor is selected. Upload the program the Arduino nano-- the serial monitor should now be used to verify a successful upload.
7. Download the Jupyter notebook software (https://jupyter.org/) and install it. Download the Arc_Analyzer_Data_Acquisition_and_Processing.ipynb file from the design files summary and move it to a file directory for data collection.
8. Place the sensors at the appropriate locations around the metal printer using the sensor clamp printed part (Fig. 6) or by other methods for a given set up.

**Fig. 6:**
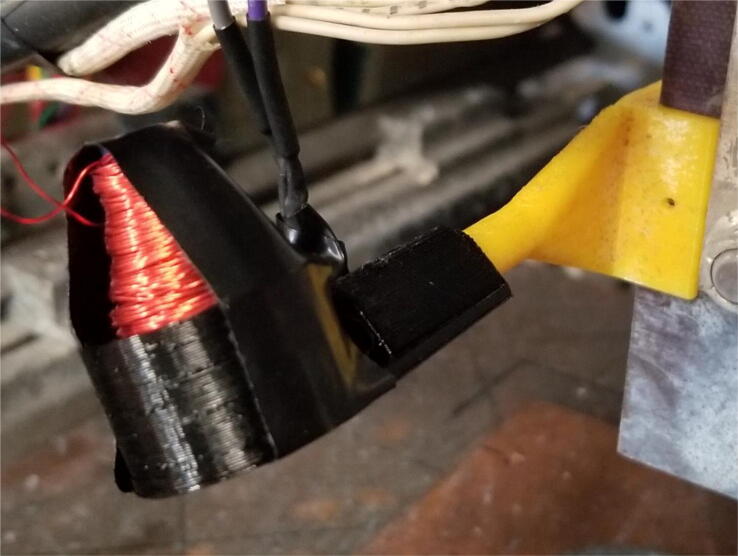
Clamping platform for radio frequency antenna and photoresistor held onto printer carriage.
9. Turn on power to the Arc Analyzer
10. Run a WAAM 3-D print and collect data.

### 5.2 Arc analyzer detailed explanation

Current, voltage, sound, radio frequency, light, and temperature sensors were used with a custom circuit board. A webcam was also used to capture video of the welding process and used for qualitative assessment. Both the analog and digital channels of the NanoDAQ are adjustable to service a wide variety of signal types and ranges. As such – the board can be assembled with all components added, and then certain sub-circuits can be bypassed by use of the on-board jumpers. In the case of the Arc Analyzer, unused sub-circuits were not soldered to the board to reduce overall costs.

**Fig. 7:**
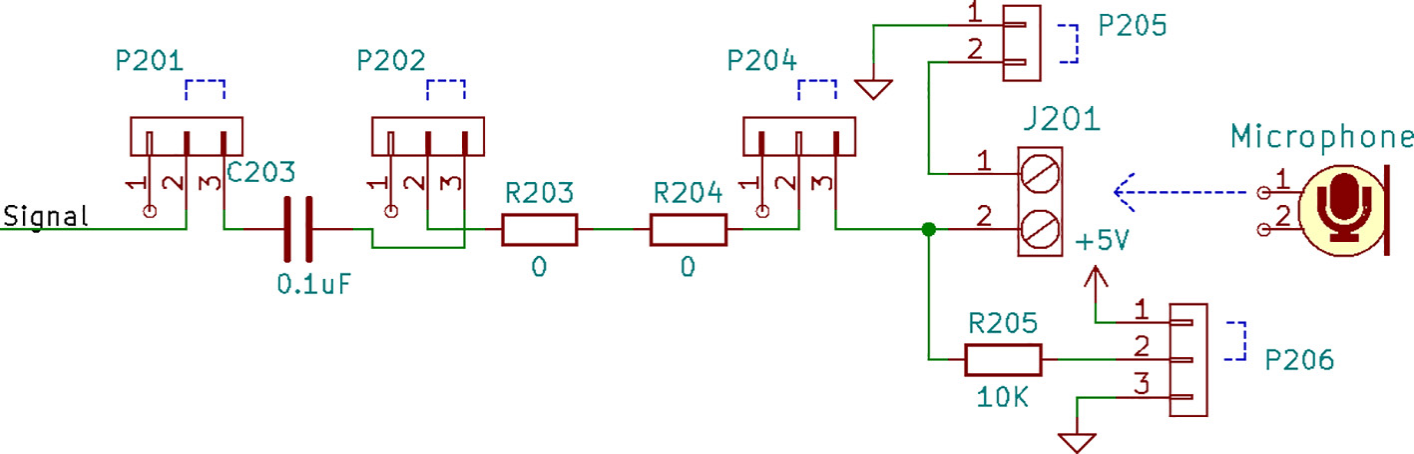
Channel 0 on the Arc Analyzer shows the microphone circuit.

Channel 0, shown in Fig. 7, on the NanoDAQ is configured to condition the signal output of a CMA-4544PF-W electret condenser microphone [33] The microphone was selected for its low cost, and acceptable sensitivity range of 20–20 kHz. The microphone is biased with a 10 KΩ resistor and then linked to a 0.1 µF ceramic capacitor for DC voltage rejection. The microphone has suitable enough gain such that an amplification circuit is not needed.

**Fig. 8:**
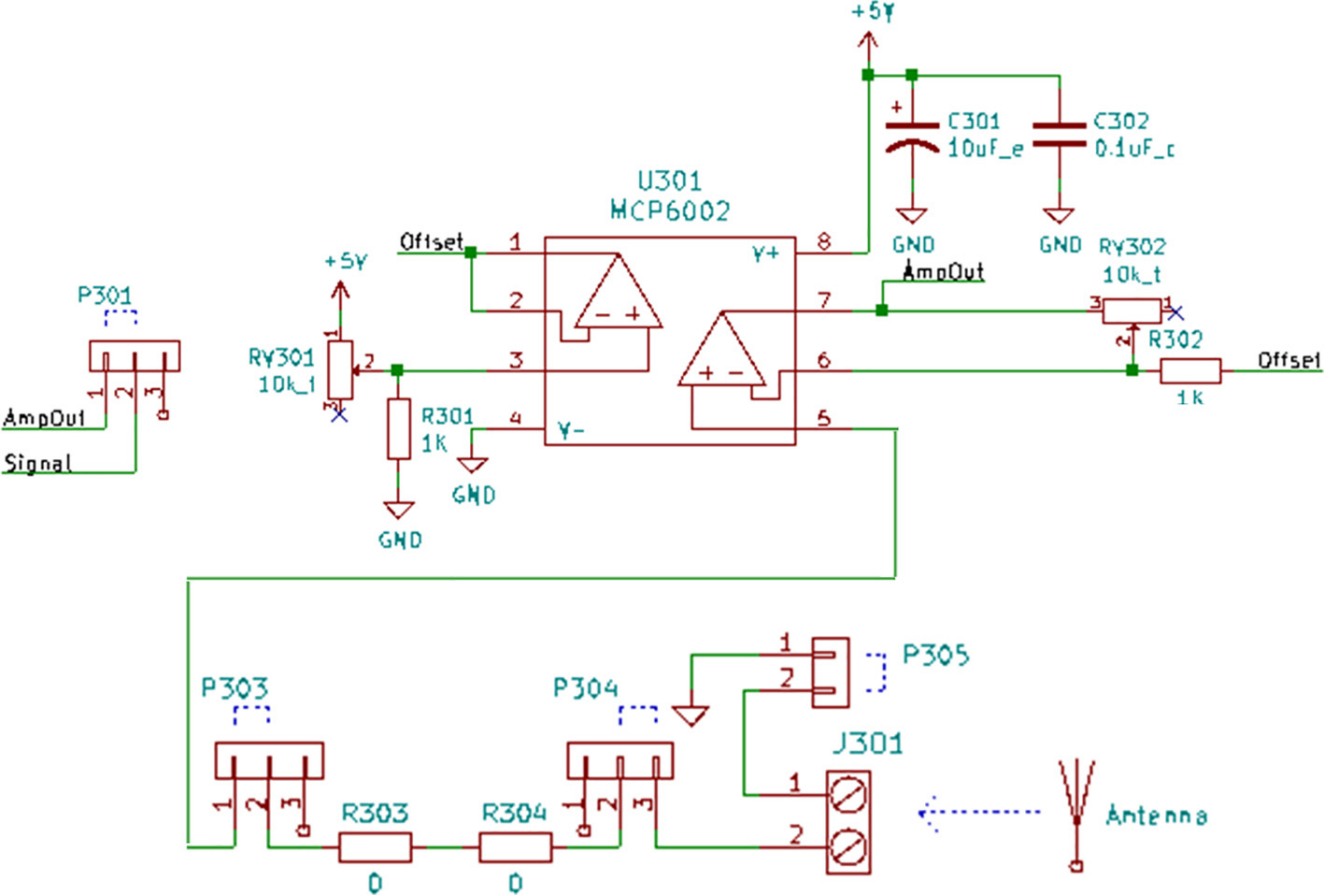
Channel 1 on the Arc Analyzer shows the radio frequency antenna circuit.

Channel 1, shown in Fig. 8, is connected to a custom antenna. The antenna consists of a PETG 3-D printed core, which is conically shaped with a maximum radius of 20 mm, and a minimum radius of 5 mm. Approximately 100 wraps of 28AWG enamel coated magnet wire are applied around the core. This is to approximate the function of a standard gain horn wave guide [34]. The dimensions of the antenna are made such that it is small and easy to position. It should be noted that this antenna design is not necessarily optimized for the low frequency expected to be emitted from the arc. This measurement is not intended to be quantitative, and instead is intended to be used at face value to compare across different parameter sets. The output of the antenna is expected to be a considerably low amplitude, and so Channel 1 is configured to implement a tunable amplifier.

**Fig. 9:**
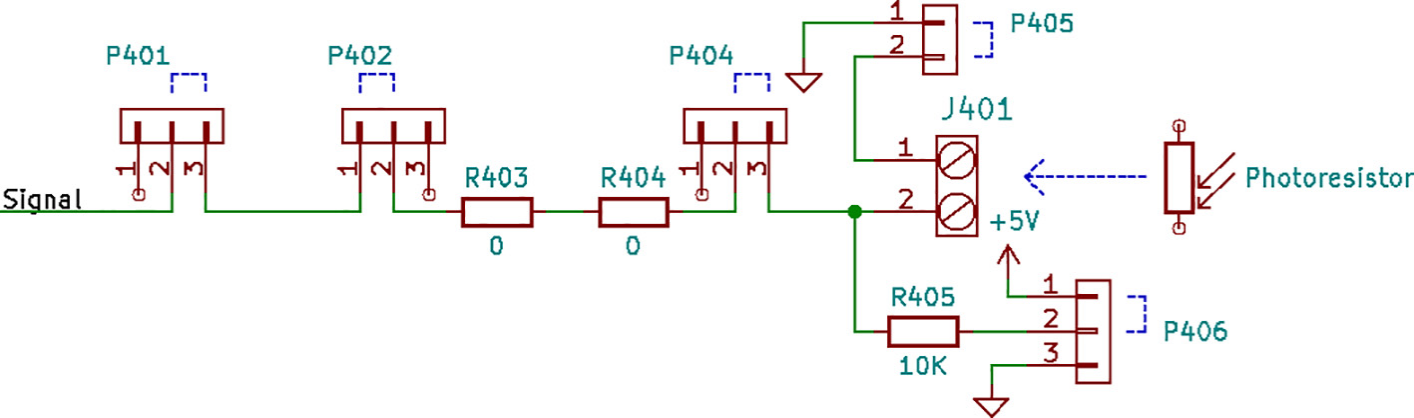
Channel 2 on the Arc Analyzer shows the photoresistor circuit.

Channel 2, shown in Fig. 9 is configured for measurements from a 10 K photoresistor. The photoresistor is put in series with a fixed 10 K resistor to create a voltage divider. The varying intermediate voltage can is measured to provide a value proportionate to the current light intensity.

**Fig. 10:**
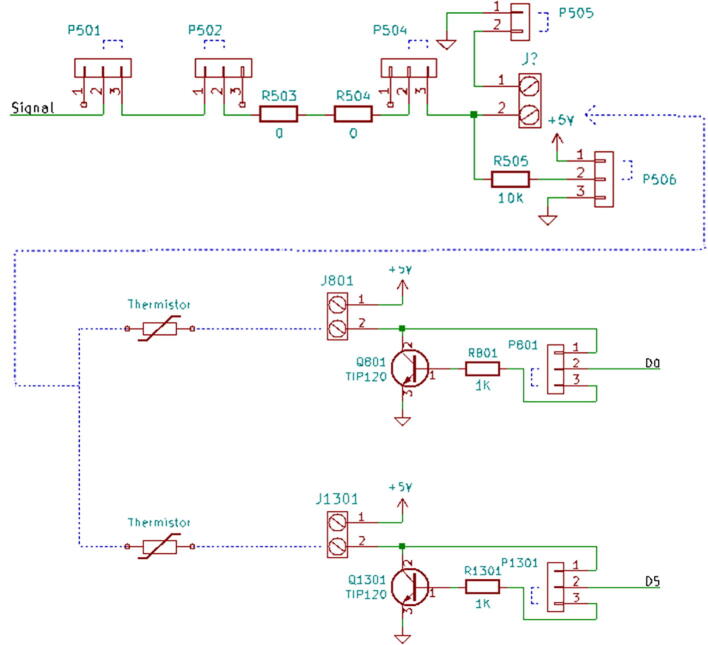
Channel 3 on the Arc Analyzer shows thermistor circuit.

Channel 3, shown in Fig. 10, is wired to handle temperature measurements. The measurement circuit itself is similar to the configuration used for Channel 2 in that it is providing a fixed 10 K resistor to complete a voltage divider made in conjunction with a 10 K 3950B thermistor [35]. To conserve analog channels, 6 identical thermistors have their ground connection hooked to one of the available digital outputs on the board. Each output channel is hooked to a TIP120 transistor which when disabled will leave the sensor floating. The entire configuration operates effectively as a signal multiplexer. Temperatures cannot be measured simultaneously with this configuration, but this is not an issue given that the Arduino Nano is not capable of parallel processing.

**Fig. 11:**
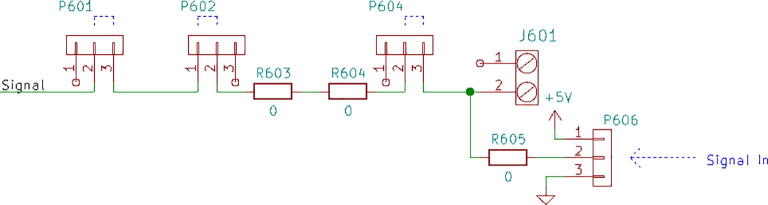
Channel 4 and 5 on the Arc Analyzer show the joining circuit with the specialized board [30].

Channel 4 and 5, shown in Fig. 11, are made to accept measurements from the welder voltage and current measurement board [30]. The board conditions the measurements to 0 to 5 volt signals. These signals are already ideal for the Arduino Nano’s ADC range. Because of this, additional conditioning is not necessary.

**Fig. 12:**
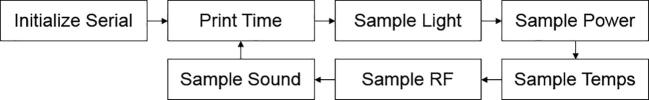
Simple looping system used by the Arc Analyzer Arduino code.

The Arduino Nano’s firmware employs a straightforward constantly measuring and looping system and is shown in Fig. 12.

First, the serial baud rate is initialized at 1,000,000 bits per second. Then the “millis()” command is invoked which gives the number of milliseconds which have elapsed since the process started. The first measurement in the loop is a single value sampled from the photoresistor. Immediately after the light intensity is measured, the reading is scaled on a range of 0–100 (100 being the most intense reading) and transmitted via serial. Next, the voltage and current are measured pseudo-simultaneously by measuring 100 samples from each channel and averaging them. These averages are immediately sent via serial. The temperatures are measured one-at-a-time, being careful to make sure to only enable on sensor at a time (as 2 sensors enabled would create drastically skewed and unusable results).

The final sets of measurements are the radio frequency (RF) and sound. These raw data sets will be later processed via Fourier transform. For both the RF and sound, 64 samples (*N*) are captured with a time interval of 60 µS (*D*) in between. Transmission of both RF and sound is delayed until all 64 samples have been acquired. With provisions for the Nyquist frequency, the minimum (*F_min_*) and maximum (*F_max_*) frequency that can be detected given this configuration are calculated in Eqs. (1), (2), respectively [36].

(1) $$F_{\mathrm{Min}}=\frac{\text{1}}{\mathrm{ND}}=\frac{\text{1}}{\text{64}\times \text{0.00006}}=\text{260}\;Hz$$

(2) $$F_{\mathrm{Max}}=\frac{\text{1}}{\text{2}D}=\frac{\text{1}}{\text{2}\times \text{0.00006}}=\text{8333}\;Hz$$

All sensors, except for current and voltage, are gathered directly by the arc analyzer circuit board via custom Arduino firmware. The data acquisition firmware communicates with the main Python program on a master personal computer over the serial port [32].

During the welding process, the main program reads data over the serial communication channel and appends it to a text file after each measurement cycle every 121 ms. Therefore, by the end of the data acquisition, an N × M array of raw data in text format is generated, where N is a number of measurement cycles during welding and M = 138 is a raw data set which could be broken down in order as timestamp, light, voltage, current, temperature (6×), radio frequency (64×), and sound (64×). The timestamp for each measurement cycle provides an average time of 121 ms.

During processing, the text file is parsed into data arrays corresponding to the measured physical parameters. Light, voltage, current, and temperature can be visualized as is or after a smoothing procedure. The data arrays with sound and radio frequency measurements have shapes of N-by-64, where N is the number of measurement cycles and each cycle consists of 64 data samples. For each of the N measurement cycles a Discrete Fourier Transform (DFT) has been applied according to the following Eq. (3):

(3) $$y_{k}=\sum_{l=\text{0}}^{L-\text{1}}e^{-\left(\text{2}\pi j\frac{\mathrm{kn}}{L}\right)}\cdot x_{l}$$

where $y_{k}$ is a DFT of the raw input L-length data sequence $x_{l}$.

According to the 0.1 ms Arduino delay between each of the 64 measured samples, the frequency range obtained after Fourier transform will be limited by equation (4):

(4) $$f_{\max}=\frac{1}{2\cdot T}=\frac{1}{2\cdot 0.1\cdot 10^{-3}}=5\cdot 10^{3}\left(\mathrm{Hz}\right)$$

where $f_{\max}$ is the maximum recorded frequency and $T=\text{0.1}\cdot \text{1}{\text{0}}^{-\text{3}}\left(s\right)$ is the time delay between data samples.

## 6 Operation instructions

### 6.1 Basic overview of data collection

The use of the Arc Analyzer requires the operation of the wire arc-based metal 3-D printer. The standard operating procedure for the WAAM setup can be found on Appropedia.org [37]. In general, after the metal substrate has been cleaned and clamped, the 3-D printer prepared and zeroed with proper gcode safely tested, the data collection can be set up. It is important to verify the data collection process before printing occurs. This is done by doing a “dry run” of the print using 0% WFS and ensuring the emergency stop is engaged, while collecting data. Inconsistencies in the collected output txt file should be noted and resolved before any further data collection.

### 6.2 Safety considerations

There are several safety warnings to consider for the safe operation of the Arc Analyzer as it is used in conjunction with a metal 3-D printer, which are presented below.

1. Electrical shock: The welding current pathway should be grounded correctly, and isolated from any other electronics.
2. Bright light: During printing, there is a plasma arc and this creates an extremely bright light, users should not look at the arc.
3. Ultraviolet light: The plasma arc generates UV light and leads to eye and skin damage. Coverings of the enclosure or welding masks are required.
4. Toxic fumes: the welding process creates toxic fumes that are dependent on the metal alloy and proper ventilation of the work-space is necessary.
5. Burn: During and after printing the work piece and work area are hot, avoid touching these areas until they have cooled.

### 6.3 Operation

1. Conduct the metal additive SOP to ensure proper set up of the metal printer.
2. Verify the position and connection of all sensors. They may have moved or become loose during previous operation.
3. Connect the Arc Analyzer to a computer via a USB cable.
4. Initialize the anaconda prompt and input code to change the file directory (cd “directory address”) used for data collection (Fig. 13). This will open the Jupyter Notebook directory.

**Fig. 13:**
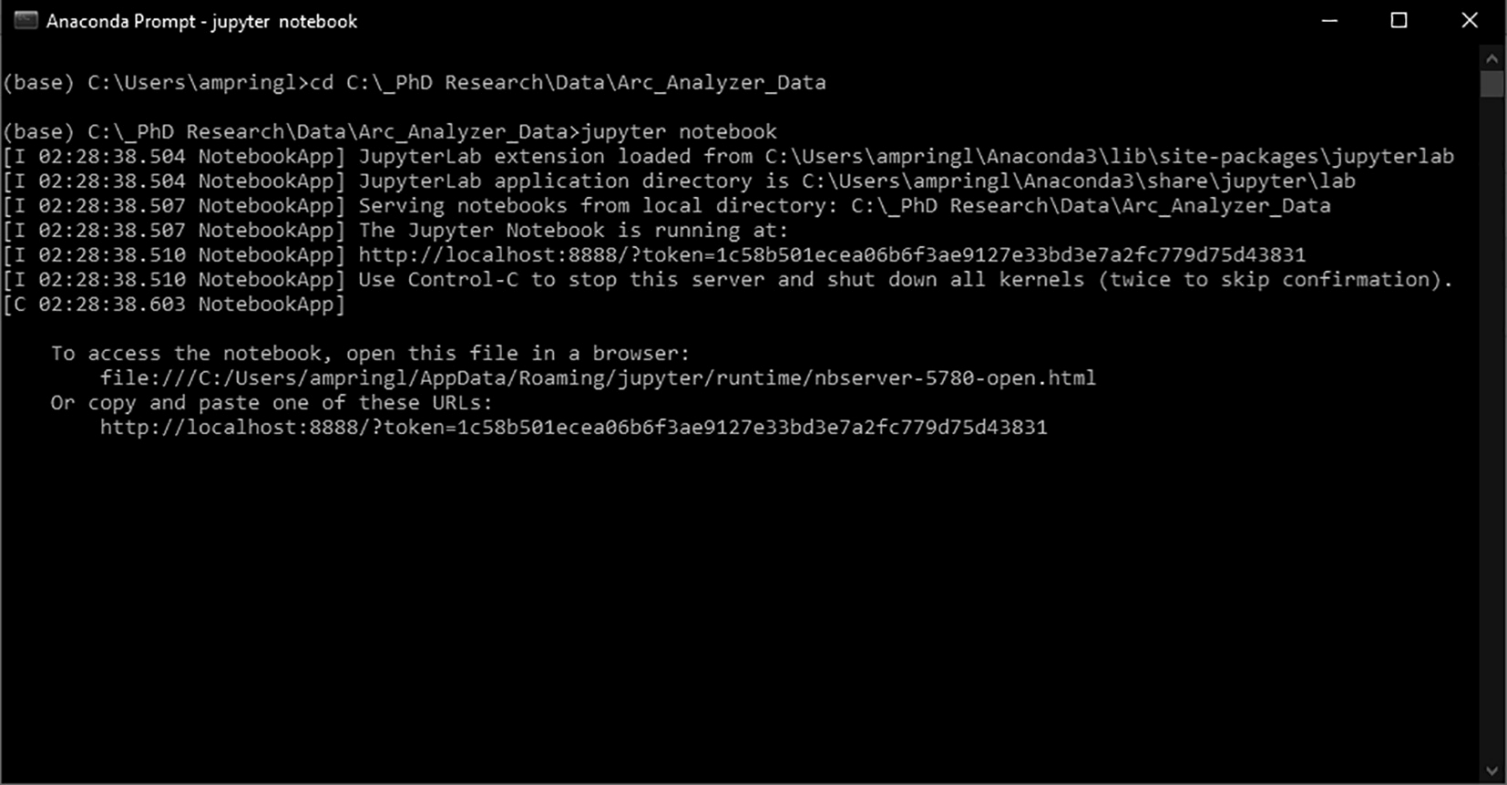
Anaconda prompt for Jupyter Notebook open-sourced web application.
5. Open the ipynb file: Arc_Analyzer_Data_Acquisition_and_Processing.ipynb.
6. Input the test duration in seconds under timer and a viable filename (Fig. 14).

**Fig. 14:**
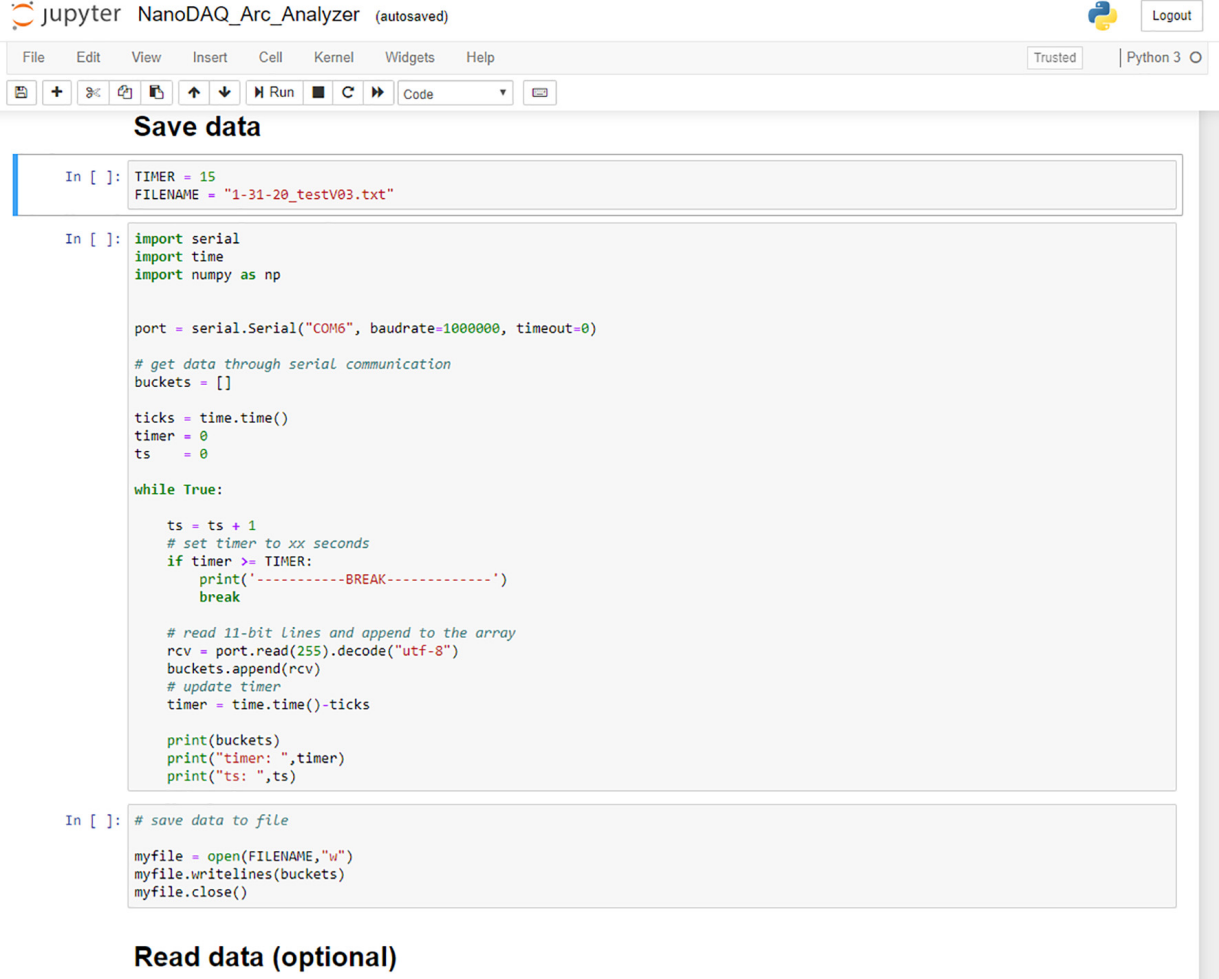
Jupyter Notebook interface detailing settings such as the measurement duration and filename designation.
7. Run all cells in the Jupyter Notebook interface while simultaneously starting the print using open source Franklin control software [38] and beginning the recording (Fig. 15).

**Fig. 15:**
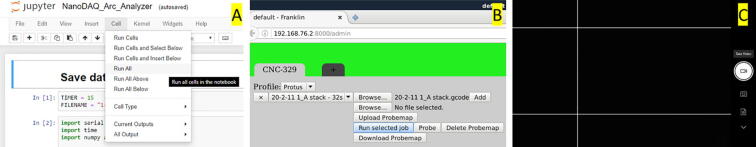
Jupyter Notebook run all command (A), Franklin printer firmware run selected job command (B), and video recording (C).
8. After the print has finished, wait for the printer to come to come to a complete stop before toggling the emergency stop. This breaks the welding circuit and eliminates the risk of accidental arcing.
9. Open the data file directory and inspect the .txt file generated for any inconsistencies with the lengths of lines (Fig. 16).

**Fig. 16:**
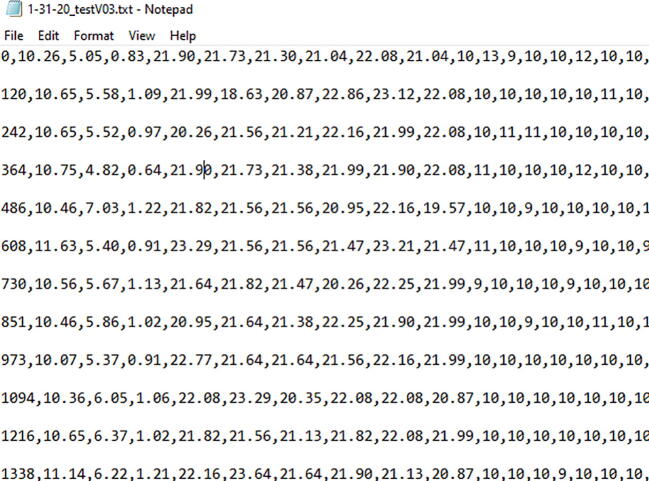
Example data output first columns of saved txt file for data analysis.
10. Call data into python or RStudio for data analysis.

## 7 Validation and characterization

### 7.1 Materials and methods

**Fig. 17:**
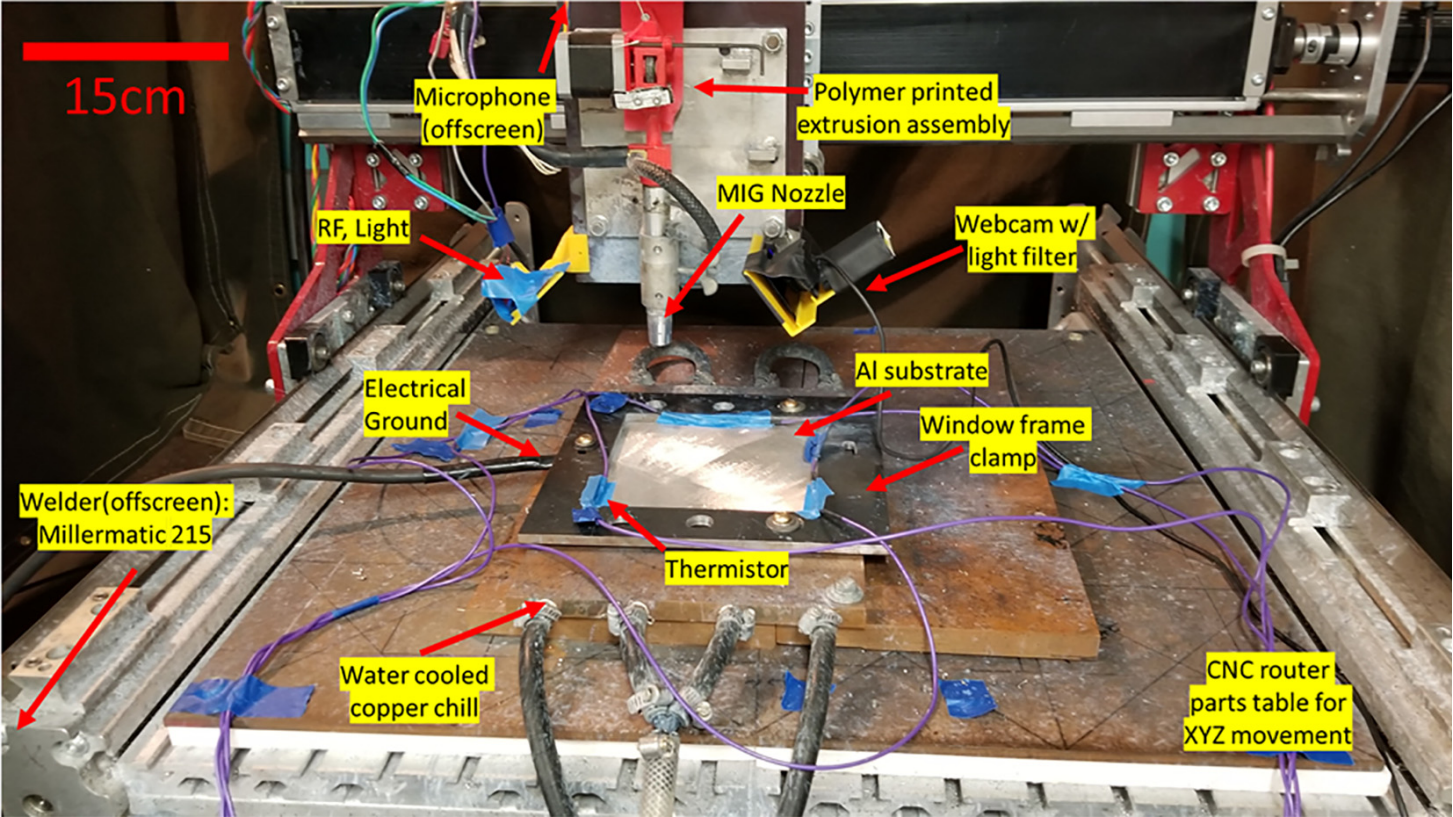
The experimental WAAM setup, scale bar of 15 cm.

In this work, a constant voltage Millermatic 190 welder was used as a power source. The build platform is a converted CNC router parts mill used for X-Y-Z control, which has been previously described by Nilsiam et al. [1]. The electrode wire was fed from a spool on top of a moving carriage down through a custom wire feed driver assembly modified from a spoolmate (Miller) gun. The experiments were carried out on a 6061-aluminum plate 300 × 300 × 2.4 mm being actively cooled via a water chilled copper plate and secured with a window frame steel clamp. The experimental set-up is shown in Fig. 17.

The electrode material of choice was 4047 aluminum due to its simple chemistry, low cost, and popular use in the welding community. The welding filler wire (Alcotec) used was 0.8 mm in diameter. Relatively thin wire was selected to both reduce the heat input necessary for the welding process and allow building on a thin base plate. The base plate material was purchased in bulk from Ryerson and then cut down to size via a plasma cutting table. The 6061 alloy was used as it is a common base material for welding. Wire and plate chemistries are shown in Table 4. The base plate was cleaned via sanding the surface oxides off with a belt sander at 400 grit until a uniform shiny surface was revealed then brushed off. The shielding gas used was 99.995% argon gas.

**Table 4:** Chemical composition of wire and base plate material.

Alloy	Si	Fe	Cu	Mn	Mg	Zn	Cr	Ti	Al
4047^a^	12	0.80	0.30	0.15	0.10	0.20	–	–	R
6061^b^	0.6	0.7	0.3	0.15	1.0	0.25	0.17	0.15	R

a: AlcoTec Wire Corporation [40].

b: Ryerson [41].

**Fig. 18:**
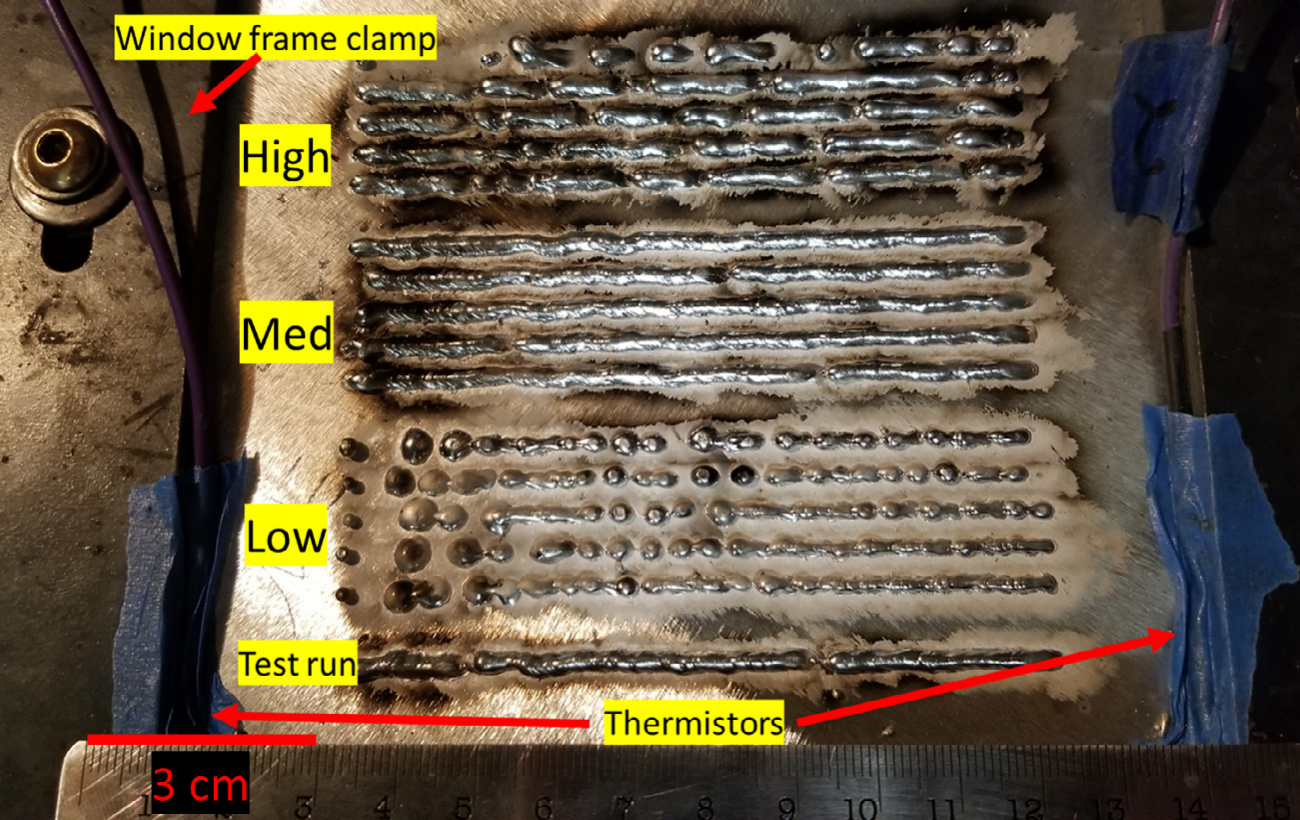
5 runs at each of the three different WFS, low, med, and high at 140, 201, and 245 mm/s, respectively. Print starts on left, and terminates on right.

The wire feed speed (WFS) of wire is the main variable considered in this work being tested at three different values (140 mm/s, 201 mm/s, and 245 mm/s) with all other parameters kept constant. The print parameters are shown in Table 5. These three WFS were selected based on an initial WFS rate exploratory study where the WFS rate was varied between 131 mm/s and 254 mm/s at intervals of 9 mm/s. The 201 mm/s run had the best consistency, lowest spatter, and weld quality. Thus, it was chosen as the ideal WFS at the set print parameters. The 140 mm/s and 245 mm/s values were selected because they were near the extremes but did not experience nozzle blockages during the exploratory study and were considered sufficently stable. For simplicity, the three WFS used will from this point on be referred to as slow, med, and high, respectively. The WFS was chosen because it is easily controlled and modified in the gcode used for tests and there is a distinct difference in depositions when varied. The gcode used generated a sample of 100 mm in length. Five runs of each WFS were deposited on the same base plate each spaced 5 mm apart within the run and 10 mm between sets. The first print of each batch started with the first run at the bottom, and fifth and final run at the top of the batch. The prints we conducted in the order of low, med, and then high WFS. Data collection was started three seconds before printing and held for three seconds after printing for each run. After printing each run, a wait time of 30 s elapsed to dissipate residual heat from the base plate before starting the next run. The samples immediately after printing are shown in Fig. 18.

**Table 5:** WAAM experimental process parameters.

Stand-off distance (mm)	Voltage (V)	Travel speed (mm/s)	Current (A)	Gas flow rate (L/min)	Wire feed rate (mm/s)
9	14.5	35	25	11.4	140 (low)201 (med)245 (high)

#### 7.1.1 Metallography and imaging

The printed lines were first cut into rectangles and then bisected transversally to reveal the microstructure normal to the weld direction at roughly the 25, 50, and 75 mm mark. Samples were then polished using silicon paper of increasing grit size: 240, 360, 420, 600, 800, with a soapy water and ethanol cleaning in between steps. Then metallography prep transitioned to steps of 6 µm, 1 µm diamond paste, then 0.05 µm colloidal silica as the final polish with soapy water, ethanol, and ultrasonic cleaning in between steps. Three runs from each WFS were then selected for metallographic analysis.

Image analysis was conducted via a python script using Jupyter Notebook utilizing optical microscopy images taken at 50× and 500× for macro and micro porosity detection, respectively. The process described below was used on 9 images for micro porosity and 15 images for macro porosity.

1. Import image as a grayscale .JPG file.
2. Crop image by determining the pixel window size to remove any edge effects, the scale bar, and non-microstructure features such as the epoxy image.
3. Use a Gaussian blur with a standard deviation of 2.5 for the Gaussian kernel.
4. Use a median filter with an 11 × 11 array of ones as the filter kernel
5. Threshold image to reveal porosity with a min area of 50 pixels and a max area of 500 kilo-pixels.
6. Set the eccentricity value to distinguish between circular, elliptical porosity. A value of 0.9 is used for 50× images, and 0.6 is used for 500× images to better distinguish solidification shrinkage vs gas induced porosity.
7. Render the composite pore image.
8. Export csv document with porosity numerical data.
9. Repeat with other images.

### 7.2 Results and discussion

The three deposited sample groups demonstrated the impact of a wide range of WFS for the selected print parameters. The printed samples are shown in Fig. 18. In general, as the individual low WFS runs progressed, the consistency of the deposition increased, transitioning from large and separated droplets with smooth texture to a thin and stable line with a more textured surface. The med WFS runs have the best weld consistent quality in appearance with only minor defects and three breaks in weld line across three different sample runs with a “stack of dimes” style texture. The high WFS runs showed large globular and separated lines with a loss of texturing resulting in smooth surfaces. A smooth solidified texture indicates an excessive droplet size before detachment where cooling rate is reduced enough to allow surface tension time to smooth the surface. In contrast, the “stack of dimes” textured surface indicates a consistent and finer droplet size being formed, detached, deposited, and a uniform solidification process. Cong et al, 2015 [42] looked at the cold metal transfer method using MIG additive on a 2××× Al alloy with single and multilayer prints. They found greater weld quality with slower travel speeds (5 mm/s to 16 mm/s tested) and lower heat input, with bead widths of about 6–8 mm. Derekar [43] completed a review of recent WAAM literature. The review is the elimination of porosity is a critical issue in welding and that the unique solidification process of WAAM techniques provides solutions to industrial needs. Ortega et al., 2018 [44] established a relationship between process parameters and deposition dimensions successfully obtaining a continuous bead size and surface quality. They demonstrate good quality depositions of up to 100 layers.

#### 7.2.1 Visual quality

Smut is micron sized metal oxide and is a byproduct of metal vapor being launched from the plasma column and exposed to the surrounding oxygen rich environment before settling down on the welded section. The smut formed is more prominently found surrounding points of excessive spatter and indicates less than ideal gas coverage. This is shown in Fig. 18 as smut is found in all runs near the beginning where the plasma arc initializes and lessens as stability is gained. Smut is minimal near the low WFS runs, present near the three arc extinction events in the med WFS runs, and greatest in the high WFS runs near line breaks.

#### 7.2.2 Raw data

**Fig. 19:**
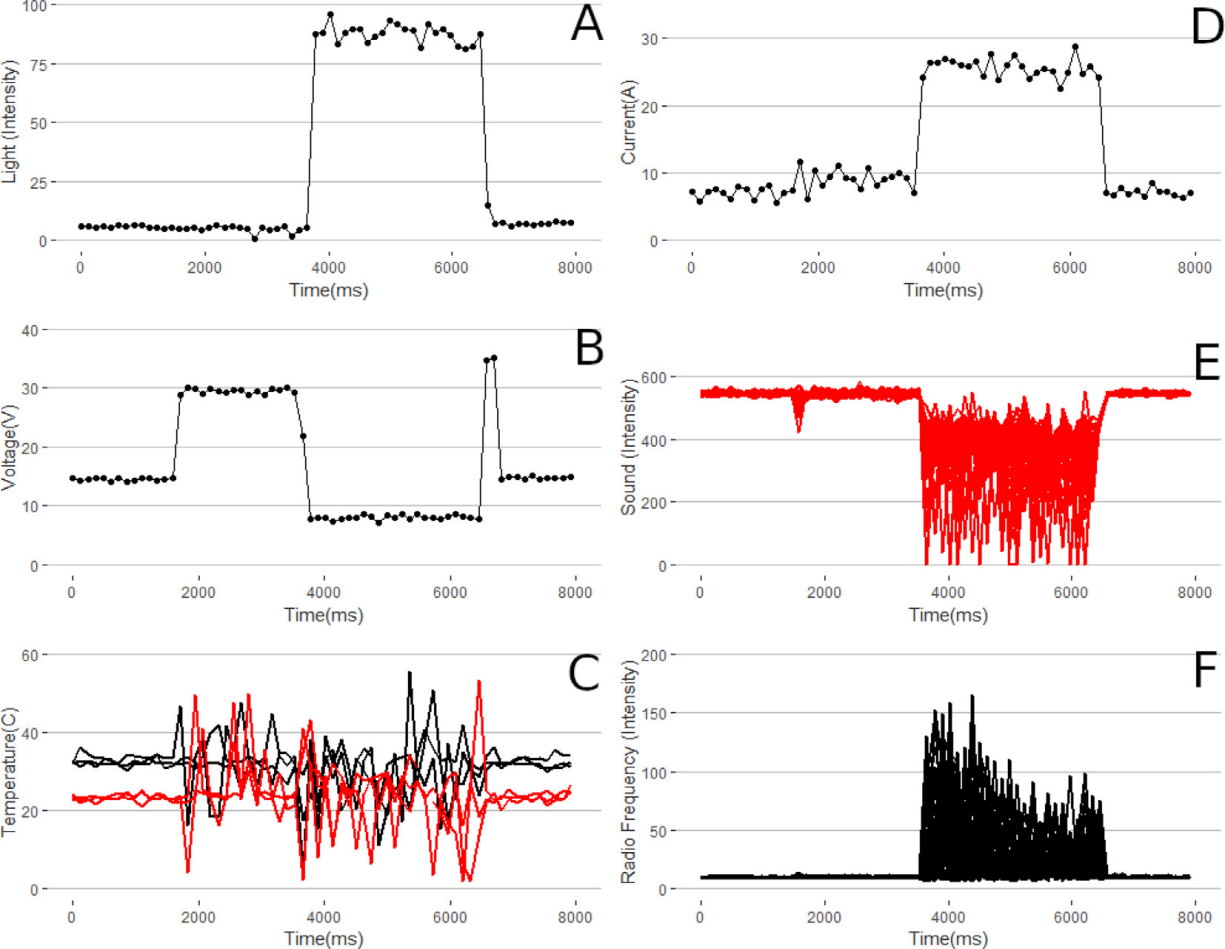
Raw data gathered via the Arc Analyzer for a given testing period. A: light intensity B: voltage, C: six thermistor readings with three off the build platform in black, and three on the build platform in red, D: current, E: raw sound, and F: raw radio frequency.

The sensor raw data output of the medium WFS third run is shown in Fig. 19. Initially, as data collection began the background voltage (19B) was measured to be 14.5 V. Two seconds before printing began the trigger relay was closed powering up the welder and allowing cover gas to flow. This is shown as a jump in the voltage level to 29 V. For these two seconds there is no WFS of wire causing charge build up on the electrode. At this point, the thermistor temperature sensors begin to detect electromagnetic noise shown in 19C at around 1800 ms as a large increase in signal noise. At the beginning of printing the wire descends and an electrical breakdown occurs between the electrode tip and the base plate. This is shown by a sharp decrease in voltage (19B), a sharp increase in current (19D) as the circuit is now complete. As the arc initiates there is a pulse of electromagnetic interference and sound pressure. This is shown by additional noise in the thermistor readings (19C) as well as large spikes in both the microphone (19E) and RF antenna (19F). For the duration of the print the photoresistor experiences reduced resistance and elevated values while the arc is stable. As the print ends, the gcode causes the wire to halt feeding while the welding power source is still powered for half a second. This causes a break to the arc while and is shown with a sharp increase in voltage (19B) temporarily before returning to nominal pre-printing value. The sound intensity variation (19E) is spikes consistently over the duration of the print, the spikes may be caused by relatively larger molten droplets of metal being flung from the consumable electrode wire. The radio frequency (19F) initially has relatively high intensity and then diminishes over the course of the print. This is may be due to the stabilization of the plasma column and should continue if a longer print was tested.

#### 7.2.3 Temperature sensor and voltage signal interaction

**Fig. 20:**
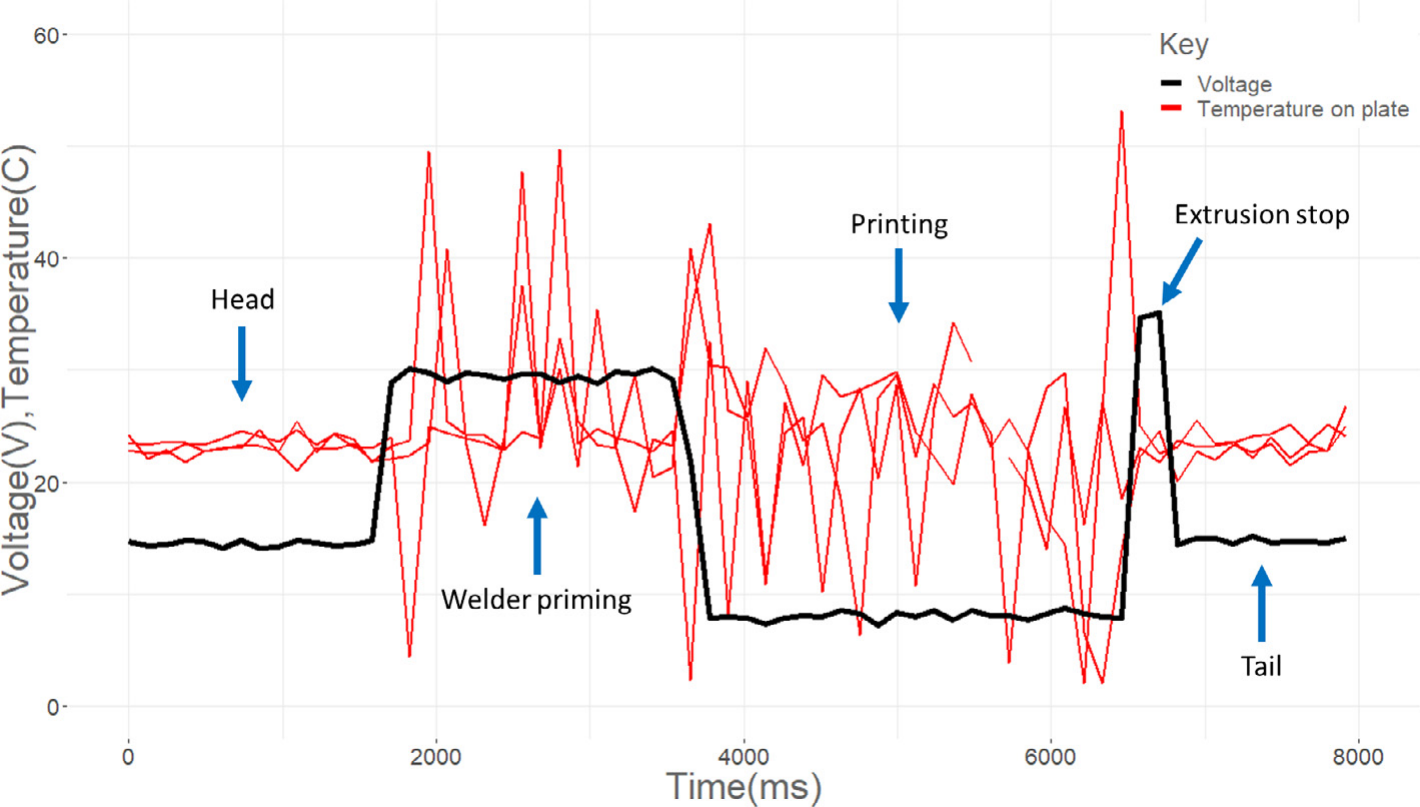
Electromagnetic interference of welding process on thermistor sensor values, voltage in red, temperature sensor in black.

The thermistor output proved to be unreliable over the printing period. Fig. 20 shows the relationship of a thermistor and the welder voltage level in four distinct regions. Initially, the voltage is stable around ~18 V and the thermistor ~22 °C. At about 1800 ms the welder is primed and a potential difference builds between the welding wire electrode and the substrate electrode. For as long as the welder is engaged, electromagnetic interference (EMI) causes the thermistor signal magnitude and noise to increase. During printing the voltage drops, and the change in EMI causes the thermistor signal to shift to a lower magnitude but maintain noise level. After printing there is a 1 s pause where wire feed stops but the welder is still engaged causing a sharp increase again in both sensor magnitudes. The thermistor output levels out as the potential difference across the electrodes is removed at the tail end of the graph similar in noise and magnitude to the head of the data. While the temperature sensors do not provide meaningful indication in line tests due to EMI, it is likely with prints lasting longer than three seconds, enough heat will build over printing to provide helpful data. This would especially be the case when comparing different print geometries or layers of different sizes or build patterns.

#### 7.2.4 Light sensor data

**Fig. 21:**
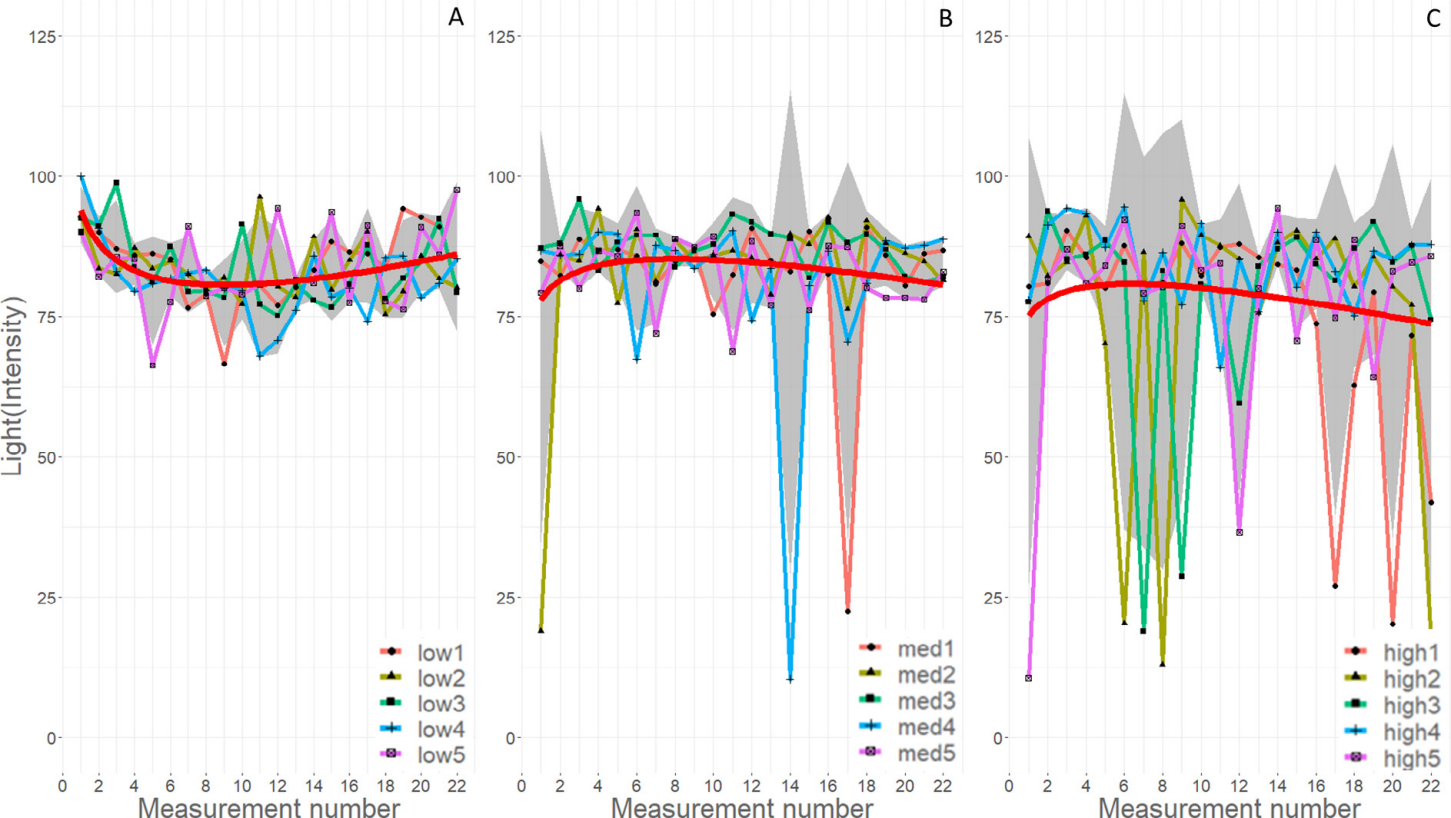
Change in photoresistor measured light intensity over the print duration for each WFS (A is low, B is med, and C is high), the red line is a smoothing formula = y ~ x + log(x), and a 95% confidence is shown in gray.

**Fig. 22:**
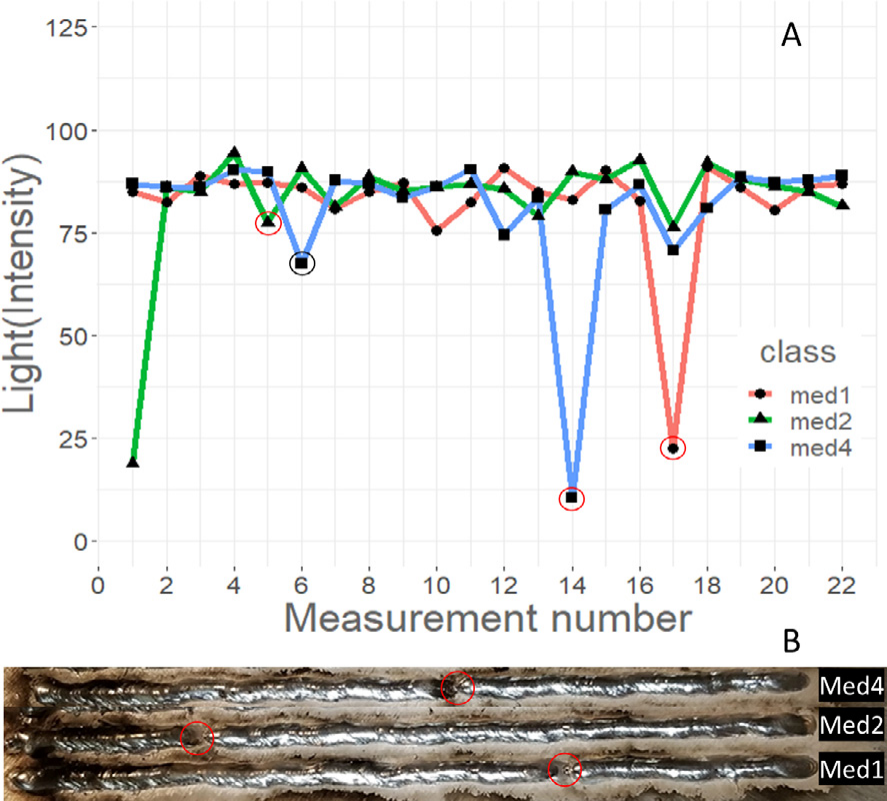
Change in photoresistor measured light intensity over the print duration for med1, med2, and med4 samples (A). Red circles match the light intensity drop with the physical defect in the weld line with the black circle representing a false drop (B).

Fig. 21 shows the measured light intensity values over the printing period for all measured samples. The light measurements over the printing period indicate when arc extinction events occur. Of the recorded data, no events were observed in the low WFS runs, two events were measured in the med WFS runs, and thirteen events were detected in the high WFS runs. There are three events for med runs observed on the samples printed, and are in three separate lines (med1, med2, and med4), shown in Fig. 22B, however, only the med1 and med4 runs were detected by the sensor and are shown in Fig. 22A. From inspection of the weld, it is likely these were points of explosive spatter due to uncontrolled short circuiting of the wire as it impacted the base plate. No arc extinction events detected in the low WFS runs is likely due to the balling up of wire material on the welding tip extending the electrode length. During the welder priming period, a voltage builds, but since there is no wire feeding and appropriate standoff distance, no arcing occurs. The standoff distance was set to 9 mm, and at that length no arcing occurs until wire feeding begins. The high WFS runs hold the most arc extinction events; however, visual inspection of the run lines indicates more than 10, line breaks. Not all major breaks in deposition line can be detected with low-light intensity measurements alone. This is most likely due to the relatively low frequency of light intensity data collection, 1 in 138 measurements every measurement number.

**Fig. 23:**
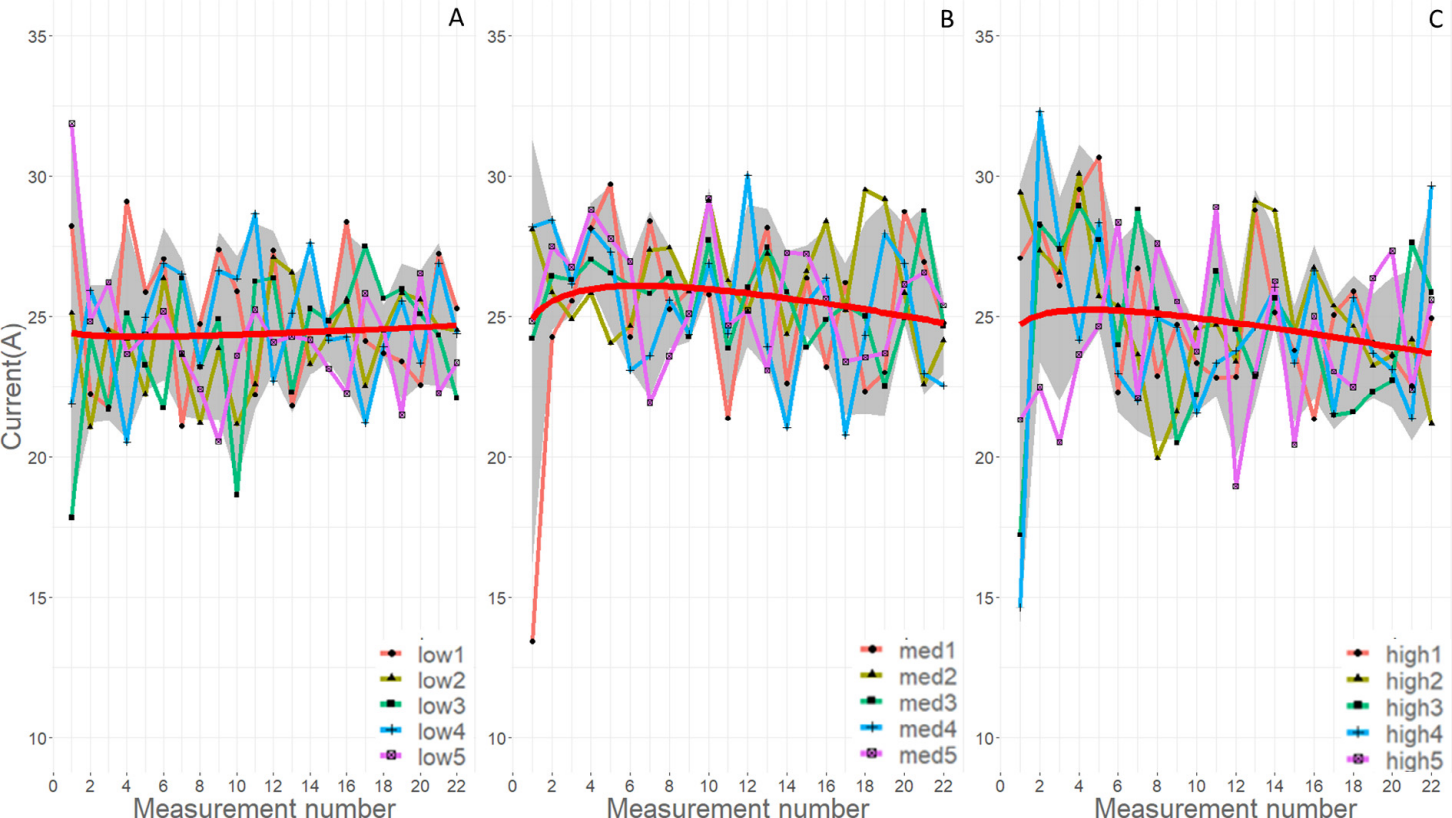
Change in measured welding current over the print duration for each WFS (A is low, B is med, and C is high), red line is a smoothing formula = y ~ x + log(x), and a 95% confidence is shown in gray.

Fig. 23 shows the measured current amperage values over the testing period for all measured samples. The amperage over the welding process is indicative of the heat generated and the consistency of the plasma arc. Any variation signifies a non-uniformity with the plasma arc conductivity and metal transfer from wire to weld pool. The general trend of the low WFS runs is a relatively consistent amperage over the print, with the med and high WFS runs having a decreasing amperage over the print duration. The slight decrease in current is likely due to the heat build up in the substrate and stabilization of the plasma arc. There is no significant difference in amperage over the different WFS rates to indicate variations in quality of weld.

**Fig. 24:**
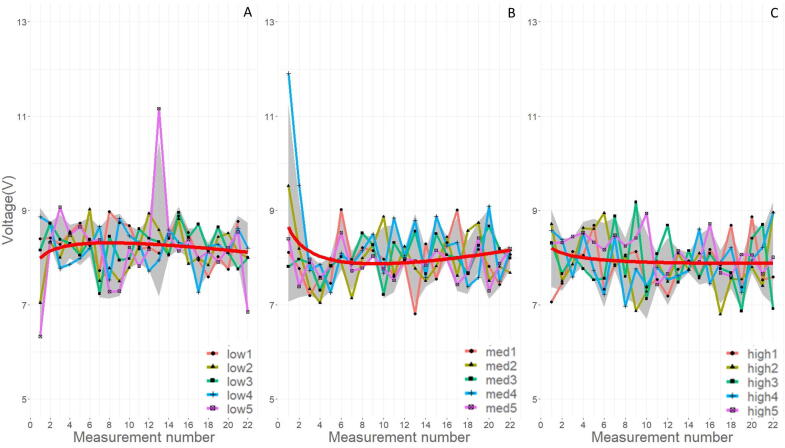
Change in measured welding voltage over the print duration for each WFS (A is low, B is med, and C is high), the red line is a smoothing formula = y ~ x + log(x), and 95% confidence is shown in gray.

Fig. 24 show the measured voltage values over the testing period for all measured samples. Voltage during the printing process signifies the arc length, the distance between the consumable electrode and the deposited material. Variation in this value indicates inconsistent mass transfer from the wire to the printed material. This variation can be visualized by differently sized molten droplets forming on the end of the metal wire and then launching towards the deposited material. The spike in in voltage for run low5 should be considered an anomaly given the testing frequency of the current method. At that measurement number, there is no coinciding spike on either the light, or current sensors. The higher values at the beginning of runs med2 and med4 are due to the sampling rate triggering during the voltage drop from the priming region to the printing region. The sampling rate for voltage is likely too slow to detect a difference in weld quality based on the WFS.

**Fig. 25:**
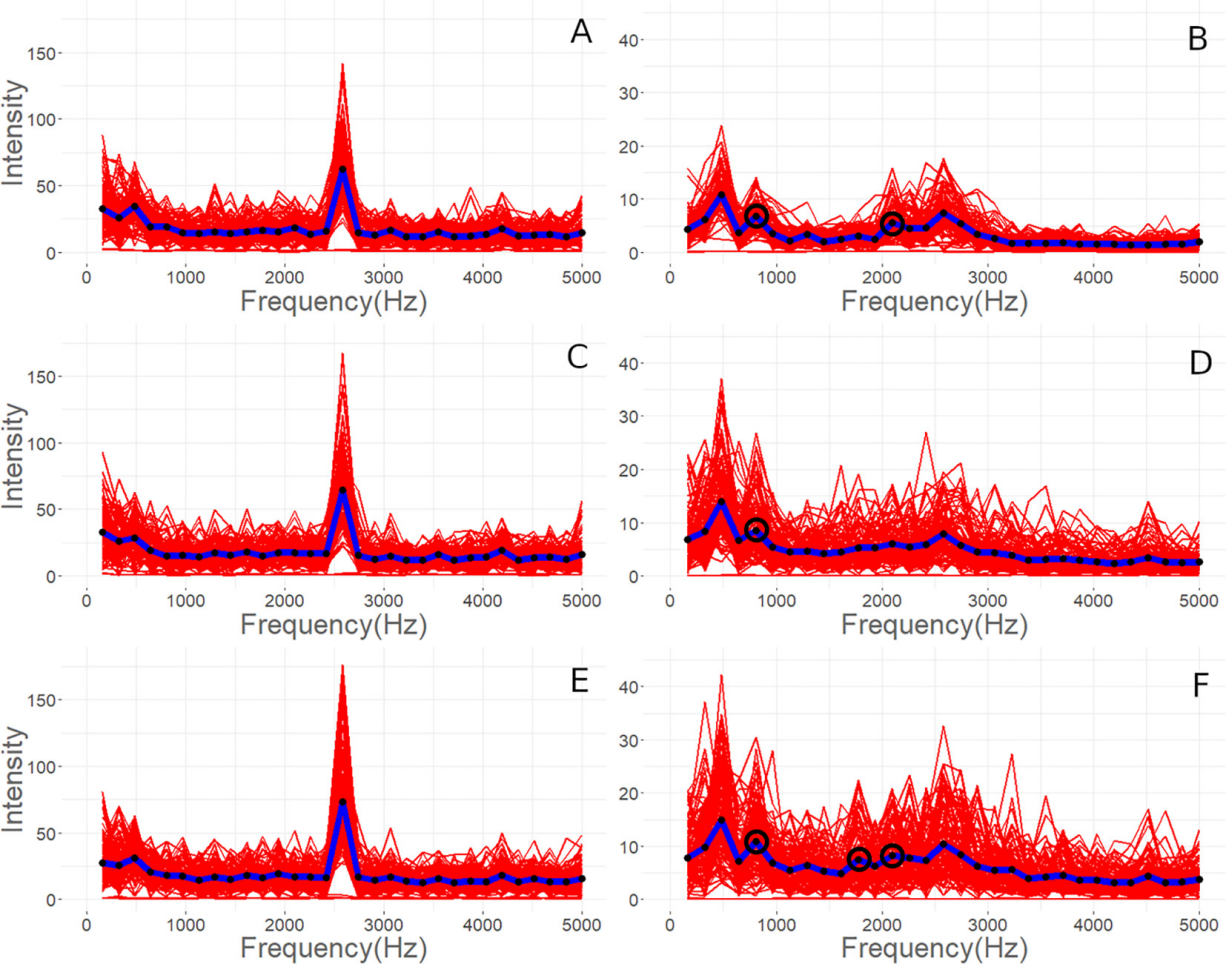
Sound FFT (A, C, and E) and RF FFT (B, D, and F) where A and B is low extrusion, C and D is med WFS, and E and F is high WFS. The blue line is the average intensity at the measured frequency.

Fig. 25 show the measured sound and radio frequency FFT values over the printing period for all measured samples. The calculated FFTs show the frequencies inherent to the welding process for the deposited samples. The sound FFT signifies a welding peak around 2600 Hz, and the general trend with increasing extrusion rate is a greater intensity of that welding peak. Akinci et al. [45] demonstrates the difference in sound FFT with regards to several welding methods and shows a characteristic peak close to 2600 Hz for MIG welding. Shahabi et al. [46] includes electrical and acoustic sensing signals for MIG quality detection, however, it generates a sound FFT with characteristic peaks lower than 600 Hz. The sound data collected is impacted by the microphone used, the MIG welding process parameters, and the mode of mass transfer. When interpreting and comparing FFT data, care must be taken to consider the differences in each process. There are no significant differences observed in the sound FFT between the different WFS, and this is likely due to the low sampling rate.

The radio frequency FTT has a few characteristic peaks, notably at 400, 800, and 2600 Hz at all WFS. The intensity of all frequency peaks increases as the WFS increases. It is interesting to note that the 400 and 2600 Hz peak are similar in frequency with several sound FFT peaks. The 800 Hz peak is not represented in the sound FFT data. The 2200 Hz peak appears in the low and high WFS runs with an additional 1800 Hz peak in the high WFS. The med WFS does not show a peak at 1800 or 2200 Hz. Radio frequency measurements in higher resolution around 1800 or 2200 Hz might be useful for a print quality metric as the lack of a peak in that region was only shown on the med samples. To the authors’ knowledge there is no literature example of radio frequency being used as a sensor for quality control in MIG welding.

#### 7.2.5 Microstructure analysis

**Fig. 26:**
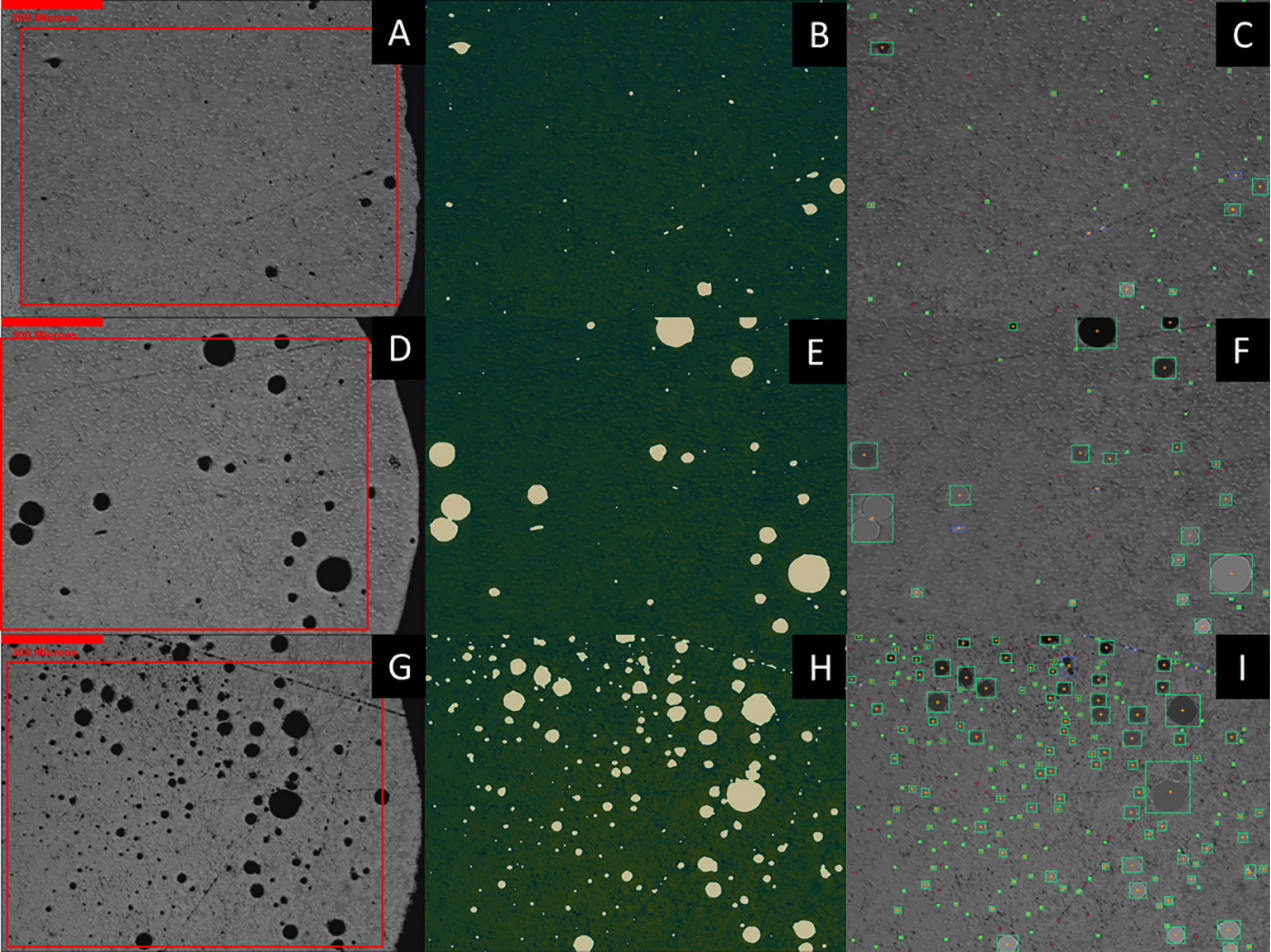
Optical microscopy of 50× magnification images of printed microstructure as-taken on left, high-contrast modified images in middle showing detected macro porosity, and fully thresholded images on right. A–C are the low WFS, D–F are the med WFS, and G–I are the high WFS.

**Fig. 27:**
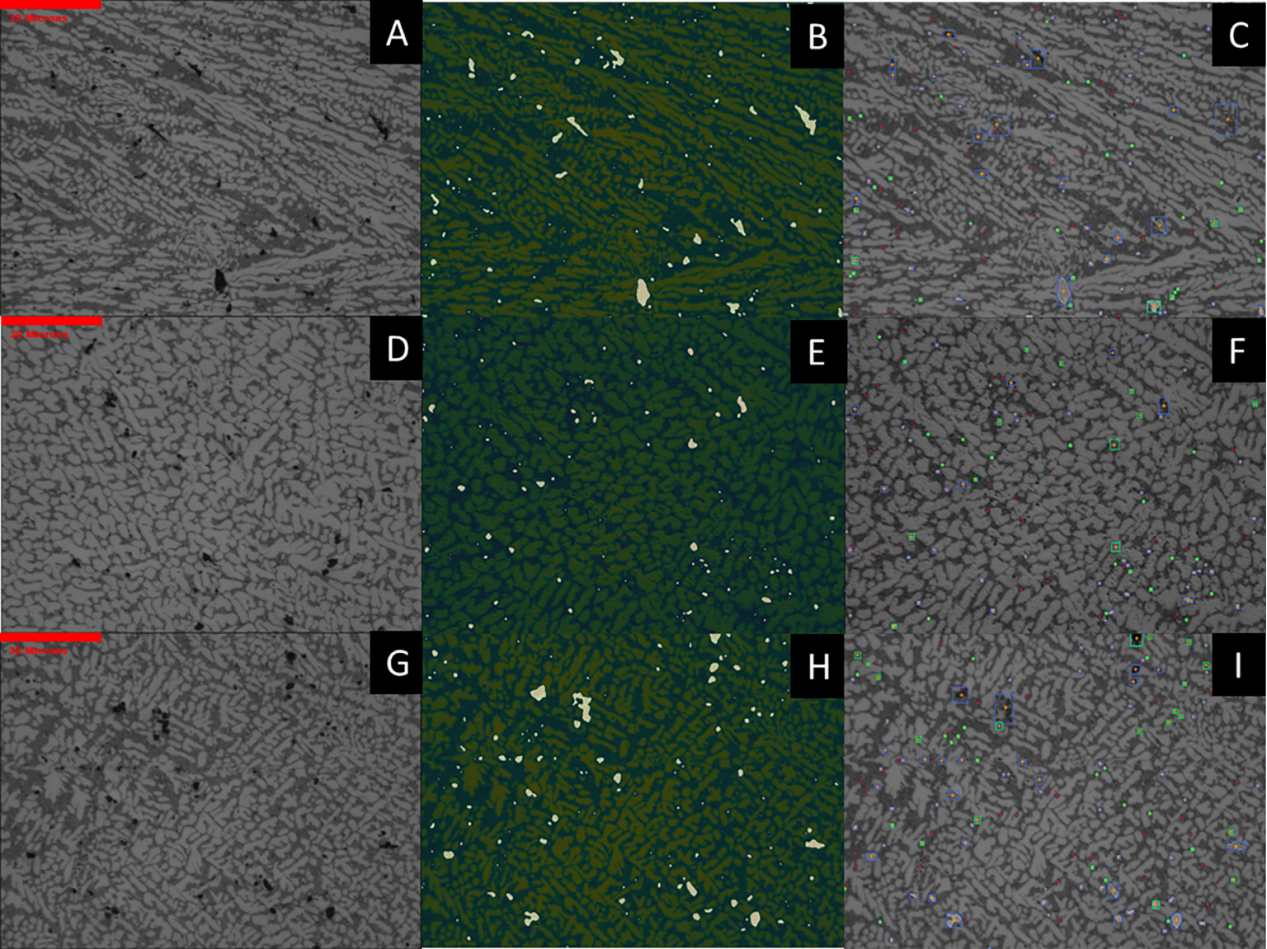
Optical microscopy of 500× magnification images of printed microstructure as-taken on left, high-contrast modified images in middle showing detected micro porosity, and fully thresholded images on right. A–C are the low WFS, D–F are the med WFS, and G–I are the high WFS.

Representative microstructures of the three WFS at 50× and 500x magnification are shown in Fig. 26, Fig. 27 with the unaltered images on the left and the modified on the right. Image analysis conducted via python script is subject to error through the image modification process described previously. Gaussian blur and median filtering were necessary to reduce the roughness of the images and works to locally average out pixel intensities (on a scale of 0–255 with 0, being black and 255 being white). This causes areas of higher contrast, allowing microstructure features such as pores to blend with the nominal microstructure and become underestimated, while polishing defects such as scratches can be eliminated completely. After the raw image is blurred and filtered, a threshold can be used to separate features of interest, both with pixel intensity as well as eccentricity, the degree of circularity. This can be seen in Fig. 26, Fig. 27 comparing the sizes and shapes of porosity between as-taken, high-contrast, and fully thresholded images. The green boxes, blue boxes, and red x’s in the fully thresholded images refer to more circular, more elliptical, and too small to be counted porosity.

The macro and micro porosity values for each WFS are shown in Table 6. As WFS increases, with equal power input and speed, greater porosity is expected as the amount of energy per length of travel remains the same as more wire mass is being forced into the plasma arc per second. This causes insufficient melting of the wire which is represented by arc extinction events of the wire impacting the print surface. Significantly more porosity percentage is observed in the high WFS sample images than both other rates. This is attributed to the more chaotic solidification of the melt pool when insufficient energy is used for consistent wire melting, causing short circuit explosions and arc extinction events. The low WFS rate runs showed more directionality and columnar dendritic growth in solidification. Due to the lower amount of metal in deposition with ample energy, undercooling was insufficient to nucleate substantial equiaxial dendrites. The med WFS micrographs show larger dendrites and dendrite arm spacings. This may be attributed to the more stable plasma arc more efficiently transferring energy to the wire and subsequently to the melt pool. From a qualitative perspective, however, more equiaxial primary aluminum dendrites were observed with less silicon-aluminum eutectic material.

**Table 6:** Macro (50×) and micro (500×) porosity determined from image analysis with 2 SE.

Extrusion Rate	Macro-porosity (%)	Macro-porosity (%)
Low	0.72 ± 0.25	0.43 ± 0.19
Med	1.70 ± 0.41	0.71 ± 0.27
High	4.39 ± 1.47	1.54 ± 0.40

**Fig. 28:**
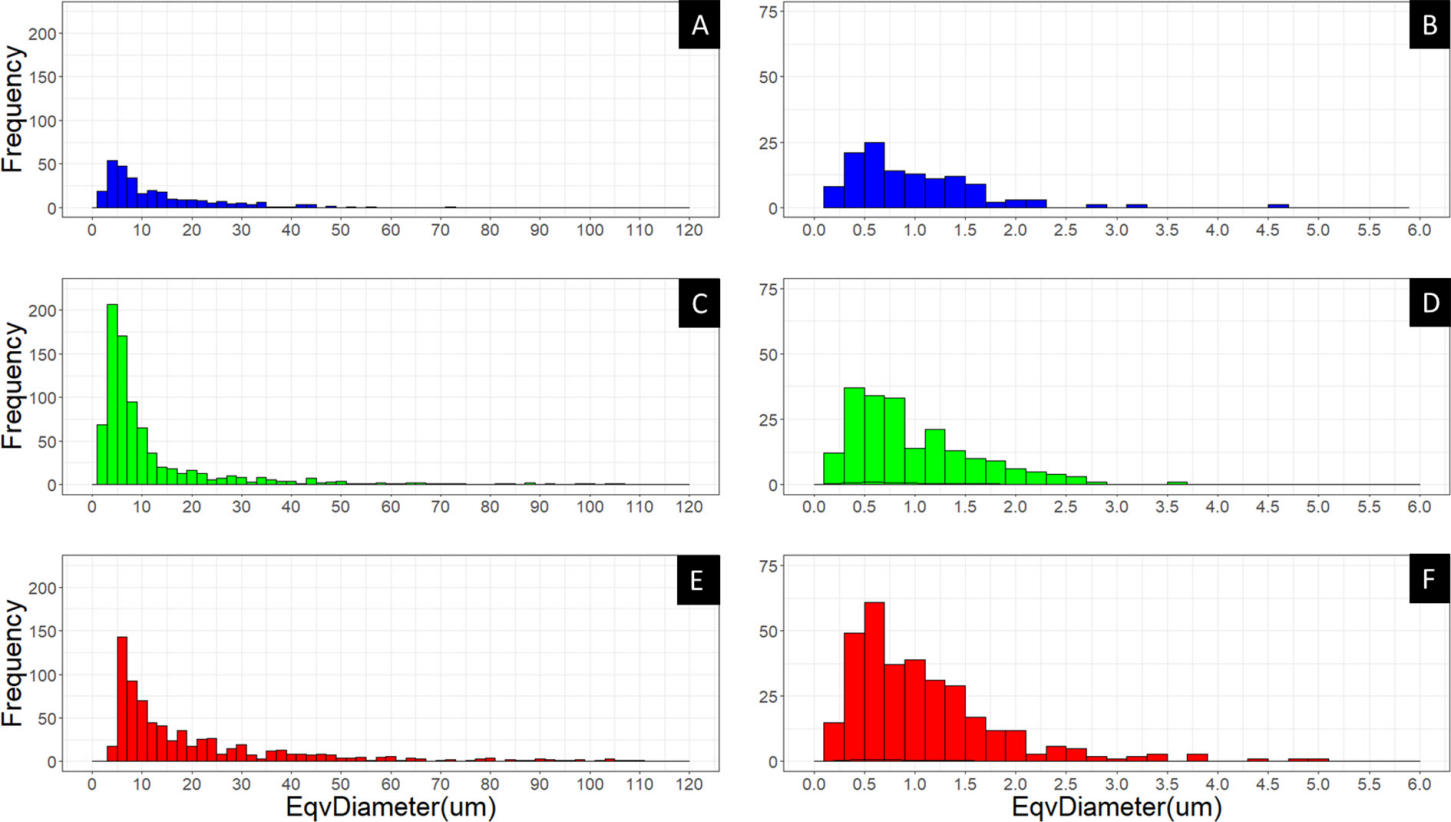
Histograms of porosity equivalent diameter, macro porosity on left, and micro porosity on right. A and B, C and D, E and F represent low (blue), med (green), and high (red) WFS respectively. (For interpretation of the references to colour in this figure legend, the reader is referred to the web version of this article.)

The larger and circular porosity observed is likely due to gas entrapment during solidification while the smaller circular porosity is due to hydrogen gas coming out of the liquid metal during solidification. The blue boxed - black irregular shapes shown in Fig. 27C-F-I are likely solidification shrinkage induced micro porosity due to lack of liquid feeding between dendrites. The placement of the shrinkage porosity would be inter-dendritic and occur in the eutectic. The general trend is an increase in porosity size, seen in Fig. 28, as the WFS increases while the other print parameters remain the same.

From the image analysis the equivalent diameter and area were determined for each detected pore as seen in Table 7. In total, 657 (330-high, 203-med, and 124-low) micro and 1832 (726-high, 820-med, and 289-low) macro pores were detected. The equivalent diameter metric allows straightforward visualization for comparing pore sizes between WFS. However, the irregularity of pore shape is ignored. Even though more macro porosity was observed in the med extrusion runs, the equivalent diameter was the smallest between the three extrusion rates. Calculation of porosity percentage was completed with the measured area of each detected pore.

**Table 7:** Macro and micro porosity image analysis derived areas and equivalent diameters.

Porosity Scale	Extrusion Rate	Count	Mean area ± 2SE (μm^2)	Total Area (μm)	Mean EqvDiameter2SE (μm)
Macro Porosity	Low	289	220 ± 60	64,439	12.7 ± 1.4
	Med	820	270 ± 60	222,788	11.6 ± 1.0
	High	726	700 ± 100	479,675	20.8 ± 1.4
Micro Porosity	Low	124	1.1 ± 0.3	132.9	0.98 ± 012
	Med	203	1.1 ± 0.2	216.5	0.99 ± 0.08
	High	330	1.4 ± 0.3	470.5	1.10 ± 0.08

Statistical analysis was completed via R-coding for both the micro and macro porosity. For both the area and equivalent diameter metrics of the macro and micro images Shapiro-Wilk normality test was conducted. Normality was rejected and the Levene’s test comparing variances and Kruskal-Wallis test comparing means and the pairwise Wilcox test were used. It is important to note that the equivalent diameter is derived from the area value determined in the image analysis.

The area and equivalent diameter metrics for micro porosity were determined to not be statistically different from each other over the three WFS. The Kruskal-Wallis test returned a p-value of 0.320 for both metrics, while the Levene’s test returned 0.147 and 0.091 for equivalent diameter and area respectively. One reason the results may not be statistically significant is that a single line may not have been a large enough volume of metal for comparison for shrinkage porosity averaging 1 µm in diameter. However, there appears to be a trend of a higher count of porosity as the WFS increases. This trend is supported by the increased variability of the light intensity (Fig. 19A–C) values and lowered trend line as WFS increases. This indicates a destabilization of the plasma arc and non-uniform heat extraction and solidification pathways leading to an increase in shrinkage porosity.

In contrast, for the macro porosity, both metrics were found to be statistically different. The Kruskal-Wallis test returned a p-value of 2.2e−16 for both metrics, and the Levene’s test returned 1.9e−14 and 3.0e−12 for equivalent diameter and area respectively. The pairwise Wilcox test revealed the largest p-value of 1.6e−4 for the low to med WFS. This signifies that there is a statistical difference in porosity as the WFS is changed. Based on the reported values in Table 6, the effect size is demonstrably large. As the porosity was six times greater in the high WFS compared to the low WFS, and more than double the med WFS. The practical importance of this relationship allows a better understanding of print parameters, how they are adjusted, and the impact that adjustment has on the solidifying microstructure.

## 8 Conclusions

The open source arc analyzer was able to monitor a low-cost gas metal arc welding process. The arc analyzer design incorporates previous work measuring current and voltage, and adds light intensity, sound, radio frequency, and temperature measurements. A series of prints was conducted varying the WFS, with sensor measurements made with the arc analyzer interpreted with R and python coding. Current and voltage variation over the printing period showed little difference between the three WFS. Arc extinction events increased as the WFS increased and were identified with the photoresistor light sensor. The temperature change of the build plate was minimal due to the short duration of prints and the location of the thermistors, but would be impactful on multi-layer or multi-line prints. The sound and radio frequency showed promise with characteristic frequencies indicating a difference between WFS, in particular the absence of an 1800 Hz peak for the med WFS, while observed for both low and high WFS runs. The most encouraging indication of a good weld is one with no arc extinction events, consistent current and voltage readings, and a lack of 1800 Hz radio frequency peak. Image analysis was conducted on metallographic cross sections to investigate porosity trends. Statistical significance was determined between the different WFS rates for macro porosity size but was not found significant for micro porosity size. Gas and shrinkage porosity increased as WFS increased while all other print parameters were constant.

## Declaration of Competing Interest

The authors declare that they have no known competing financial interests or personal relationships that could have appeared to influence the work reported in this paper.
